# Estimating dynamic vascular perfusion based on Er-based lanthanide nanoprobes with enhanced down-conversion emission beyond 1500 nm

**DOI:** 10.7150/thno.65771

**Published:** 2021-10-11

**Authors:** Qian Jia, Zheng Li, Mingli Bai, Haohao Yan, Ruili Zhang, Yu Ji, Yanbin Feng, Zuo Yang, Zhongliang Wang, Jianxiong Li

**Affiliations:** 1 Engineering Research Center of Molecular and Neuro-imaging of ministry of education, School of Life Science and Technology, Xidian University, Xi'an, Shaanxi, 710126 China.; 2 Academy of Advanced Interdisciplinary Research, Xidian University, Xi'an, Shaanxi, 710071, China.; 3 Department of Radiotherapy, Chinese PLA General Hospital, Beijing, 100071, China.

**Keywords:** Lanthanide based nanoparticles, NIR-IIb imaging, vascular imaging, peripheral artery disease, ischemic reperfusion

## Abstract

Peripheral artery disease (PAD) is a common, yet serious, circulatory condition that can increase the risk of amputation, heart attack or stroke. Accurate identification of PAD and dynamic monitoring of the treatment efficacy of PAD in real time are crucial for optimizing therapeutic outcomes. However, current imaging techniques do not enable these requirements.

**Methods:** A lanthanide-based nanoprobe with emission in the second near-infrared window b (NIR-IIb, 1500-1700 nm), Er-DCNPs, was utilized for continuous imaging of dynamic vascular structures and hemodynamic alterations in real time using PAD-related mouse models. The NIR-IIb imaging capability, stability, and biocompatibility of Er-DCNPs were evaluated *in vitro* and *in vivo*.

**Results:** Owing to their high temporal-spatial resolution in the NIR-IIb imaging window, Er-DCNPs not only exhibited superior capability in visualizing anatomical and pathophysiological features of the vasculature of mice but also provided dynamic information on blood perfusion for quantitative assessment of blood recovery, thereby achieving the synergistic integration of diagnostic and therapeutic imaging functions, which is very meaningful for the successful management of PAD.

**Conclusion:** Our findings indicate that Er-DCNPs can serve as a promising system to facilitate the diagnosis and treatment of PAD as well as other vasculature-related diseases.

## Introduction

Peripheral artery disease (PAD), a major manifestation of atherosclerosis, is defined by the narrowing or blockage of arteries that supply blood to the lower limbs 1,2. Consequently, PAD is not only the leading cause of critical limb ischemia and amputation but is also associated with a significantly increased risk of coronary or cerebrovascular diseases, such as heart attacks and stroke, affecting more than 200 million people worldwide 3-5. Despite its high prevalence and its effect on cardiovascular morbidity and mortality, PAD remains largely underdiagnosed and undertreated as it is often asymptomatic or discovered at an advanced stage, which markedly prevents patients from accessing effective treatment and leads to life-threatening conditions 6-9. Therefore, accurate detection and timely treatment of PAD are very important for preventing the worsening of the disease.

Rapid developments in medical imaging techniques, including laser Doppler imaging (LDI), computed tomography angiography (CTA), magnetic resonance angiography (MRA), and positron emission tomography (PET), have provided opportunities to obtain insights into the pathophysiological and anatomical characterization of PAD 10-13. However, most current techniques are limited by their low spatial resolution, unfavorable sensitivity, high cost, or ionizing radiation. Furthermore, in addition to the precise diagnosis of PAD, dynamic monitoring of treatment efficacy in real time plays a significant role in determining the final outcome of the treatment by maximizing treatment effectiveness while minimizing adverse effects 14-15. In fact, there are an increasing number of therapeutic options that require the guidance of imaging tools. Unfortunately, almost none of the current imaging modalities used for PAD detection is particularly effective at providing quick real-time feedback to allow valuable non-invasive assessments during treatment 16-18. Therefore, it is highly desirable to develop a robust approach that can integrate both diagnostic and therapeutic imaging functions to non-invasively assess the progression of PAD and the treatment response with higher sensitivity and resolution.

Fluorescence imaging in the second near-infrared (NIR-II) window (1000-1700 nm) holds great promise for diverse biomedical applications owing to its considerable advantages over traditional visible and first near-infrared (NIR-I, 700-900 nm) imaging, such as substantially reduced photon scattering and low tissue autofluorescence, which afford deeper tissue penetration and higher spatial resolution 19-29. Although Mie theory indicates that imaging at longer wavelengths can achieve low background and high resolution, the absorption peak of water molecules in biological tissues near 1450 nm considerably attenuates luminescence at these wavelengths and reduces penetration depth 30. Therefore, avoiding the water absorption wavelength and acquiring images in the 1500-1700 nm (referred to as NIR-IIb window) is more ideal for fluorescence imaging *in vivo* with a better signal-to-background ratio (SBR) due to nearly zero auto-fluorescence in this region 31,32. In the past decades, several NIR-IIb probes, including single-walled carbon nanotubes (SWNTs), quantum dots (QDs), small molecular dyes, Er^3+^-doped rare earth nanoparticles (Er-RENPs), metal-based nanoparticles, and semiconducting polymer dots (Pdots) have been developed for *in vivo* bioimaging 33-37. Despite significant advances, very limited inorganic NIR-IIb imaging probes beyond 1500 nm have been investigated for PAD visualization and treatment evaluation 38.

Herein, we present a multifunctional imaging strategy, which integrates the diagnostic and therapeutic imaging functions synergistically to enable real-time imaging of dynamic vascular structure changes and hemodynamic alterations in the PAD mouse models by using an NIR-IIb probe (Er-DCNPs) based on an Nd^3+^ sensitizing and Ce^3+^-doping system. Down-conversion (DC) luminescence of Er-DCNPs at 1530 nm was boosted by the Er^3+^/Ce^3+^ co-doped strategy, which highly suppressed the up-conversion (UC) pathway, induced by the electronic transition of ^4^I_11/2_ → ^4^I_13/2_ of Er^3+^, substantially enhancing the emission at 1530 nm 39. Our results demonstrated that Er-DCNPs not only exhibited superior fluorescence imaging capability in visualizing the vasculature of mice with different degrees of hindlimb ischemia but also enabled the quantitative estimation of physiological indicators (heartbeats and respiratory rate) of mice based on an imaging frame rate. Most importantly, our Er-DCNPs probe can provide dynamic information on blood perfusion for the quantitative assessment of the degree of recovery from hindlimb ischemia, suggesting its potential for the diagnosis and treatment evaluation of PAD and other vasculature-related diseases.

## Materials and Methods

### Reagents and instrumentation

All chemical reagents, if not specified, were purchased from Alfa Aesar or Sigma-Aldrich and used without purification. Methoxy-polyethyleneglycol amine (mPEG-NH_2_, MW: 5000) was purchased from Xi'an Ruixi Biological Technology Co., Ltd. (Xi'an, China). Deionized water (DI water) was used in all experiments. Murine mammary carcinoma cell line 4T1 was obtained from Shanghai Zhong Qiao Xin Zhou Biotechnology Co., Ltd. (Shanghai, China). Penicillin streptomycin, RPMI 1640 Medium, bovine serum (FBS) and 0.25% (w/v) trypsin solution were purchased from Gibco Life Technologies (AG, Switzerland). Phosphate-Buffered Saline (PBS) was purchased from OpticsPlanet, Inc. (USA). C57BL/6 female mice and BALB/c female mice (6-7 weeks old and weighing 18-22 g) were supplied by the Animal Center of the Fourth Military Medical University (Xi'an, China). Animal protocols related to this study were reviewed and approved by the Institutional Animal Care and Use Committee of the Fourth Military Medical University (approval number: 20180304). Transmission electron microscopy (TEM) images were obtained using a JEM-2100F electron microscope. The absolute quantum yield was obtained using a Fluorolog-QM (HORIBA). NIR-II live animal imaging system (Series II 900/1700-H, China) and home-built small animal NIR-II imaging system by InGaAs-NIRvana640LN camera were applied for *in vivo* imaging.

### Synthesis of NaYbF_4_:2%Er,2%Ce nanoparticles

In a typical synthetic procedure of NaYbF_4_:2%Er,2%Ce, 1 mmol of CF_3_COONa, 0.96 mmol of Yb(CF_3_COO)_3_, 0.02 mmol of Er(CF_3_COO)_3_ and 0.02 mmol of Ce(CF_3_COO)_3_ were mixed with oleic acid (10 mmol), oleylamine (10 mmol) and 1-octadecene (20 mmol) in a two-neck reaction flask. The slurry mixture was heated to 120 °C under vacuum for 30 min to remove water and oxygen. Then the solution was heated to 300 °C with a rate of 10 °C/min under dry argon flow, and then maintained at 300 °C for 60 min with stirring. After cooling to room temperature, an excess amount of ethanol was poured into the solution. The resultant nanoparticles were centrifuged at 5,000 r.p.m. for 20 min. After centrifugal washing with hexane/ethanol, NaYbF_4_:2%Er,2%Ce nanoparticles were re-dispersed in 5 mL of hexane for further coating.

### Synthesis of NaYbF_4_:2%Er,2%Ce@NaYbF_4_ nanoparticles

Typically, 5.0 mL of as-prepared NaYbF_4_:2%Er,2%Ce solution, 1 mmol of CF_3_COONa, 1 mmol of Yb(CF_3_COO)_3_ were mixed with oleic acid (20 mmol) and 1-octadecene (20 mmol) in a two-neck reaction flask. Then the mixture was heated to 120 °C under vacuum for 30 min to remove hexane and water. The flask was then heated to 305 °C for 75 min with stirring. After cooling to room temperature, an excess amount of ethanol was poured into the solution. The resultant nanoparticles were centrifuged at 5,000 r.p.m. for 20 min. After centrifugal washing with hexane/ethanol, NaYbF_4_:2%Er,2%Ce@NaYbF_4_ nanoparticles were re-dispersed in 5 mL of hexane for further coating.

### Synthesis of NaYbF_4_:2%Er,2%Ce@NaYbF_4_@NaNdF_4_:50%Yb nanoparticles

The synthetic procedure of NaYbF_4_:2%Er,2%Ce@NaYbF_4_@NaNdF_4_:50%Yb was the same as that used to synthesize NaYbF_4_:2%Er,2%Ce@NaYbF_4_ nanoparticles, except that 5 mL of as-prepared NaYbF_4_:2%Er,2%Ce@NaYbF_4_ solution, 1 mmol of CF_3_COONa, 0.5 mmol of Yb(CF_3_COO)_3_ and 0.5 mmol of Nd(CF_3_COO)_3_ were mixed with oleic acid (20 mmol) and 1-octadecene (20 mmol) in a two-neck reaction flask. The as-synthesized NaYbF_4_:2%Er,2%Ce@NaYbF_4_@NaNdF_4_:50%Yb nanoparticles were re-dispersed in 5 mL of hexane for further coating.

### Synthesis of NaYbF_4_:2%Er,2%Ce@NaYbF_4_@NaNdF_4_:50%Yb@NaLuF_4_ nanoparticles

The synthetic procedure of NaYbF_4_:2%Er,2%Ce@NaYbF_4_@NaNdF_4_:50%Yb@NaLuF_4_ was the same as that used to synthesize NaYbF_4_:2%Er,2%Ce@NaYbF_4_@NaNdF_4_:50%Yb nanoparticles, except that 5 mL of as-prepared NaYbF_4_:2%Er,2%Ce@NaYbF_4_@NaNdF_4_:50%Yb solution, 1 mmol of CF_3_COONa, 1 mmol of Lu(CF_3_COO)_3_ were mixed with oleic acid (20 mmol) and 1-octadecene (20 mmol) in a two-neck reaction flask. The as-synthesized NaYbF_4_:2%Er,2%Ce@NaYbF_4_@NaNdF_4_:50%Yb@NaLuF_4_ nanoparticles were re-dispersed in 10 mL of chloroform for further modification.

### NaYbF_4_:2%Er,2%Ce@NaYbF_4_@NaNdF_4_:50%Yb@NaLuF_4_@PMH-COOH preparation

80 mg of poly 1-octadecene-maleic anhydride (PMH) was dissolved in 1 mL of chloroform in 5 mL flask, and then 1 mL NaYbF_4_:2%Er,2%Ce@NaYbF_4_@NaNdF_4_:50%Yb@NaLuF_4_ in chloroform was added dropwise, followed by 12 h of stirring at room temperature. Subsequently, the round-bottomed flask was placed in a vacuum drying oven and maintained at 50 °C for 5 h. 2 mL of purified water and 80 mg of 4-Dimethylaminopyridine (DMAP) was then added to above flask under ultrasonic irradiation until the solution was clear and transparent. After centrifugation at 14,000 rpm for 1 h, the excess surfactant was removed to obtain hydrophilic nanoparticles NaYbF_4_:2%Er,2%Ce@NaYbF_4_@NaNdF_4_:50%Yb@NaLuF_4_@PMH-COOH, which were dissolved in 5 mL of 2-(N-morpholino)ethanesulfonic acid (MES) buffer (pH 8.5) for later use.

### NaYbF_4_:2%Er,2%Ce@NaYbF_4_@NaNdF_4_:50%Yb@NaLuF_4_@PMH-mPEG5000 probe preparation

1 mL of NaYbF_4_:2%Er,2%Ce@NaYbF_4_@NaNdF_4_:50%Yb@NaLuF_4_@PMH-COOH solution was added into 2 mL of MES buffer (pH 8.5), and then 2 mg of mPEG5000-NH_2_ dissolved in 1 mL of MES buffer (pH 8.5), 2 mg EDC (1-(3-dimethylaminopropyl)-3-ethylcarbodiimide hydrochloride) dissolved in 1 mL MES buffer (pH 8.5) was slowly dropped into the above reaction solution, and reacted at 37 °C for 12 h. Then the reaction solution was extensively dialyzed against PBS for 3 h to remove excess reactants and by products. Finally, the reaction solution was centrifuged at 4000 r.p.m. for 10 min to remove the larger particles, and the upper solution was concentrated with an ultrafiltration tube to obtain the target probe, which is denoted as Er-DCNPs.

### Fluorescence imaging of the mice

Before imaging, the hair of all the mice was removed by using hair clipper and depilatory cream. The mice were intravenously injected with 100 µL probes. Fluorescence images were acquired by Series II 900/1700-H. All the fluorescence photos were gathered by a 640 × 512 pixel InGaAs-NIRvana640LN camera. An 808 nm laser diode was decided to be the excitation laser for both of the contrast agents at a power density of 400 mW/cm^2^ (lower than the safe exposure limit of 1.0 W/cm^2^ determined by the International Commission on Nonionizing Radiation Protection). A 1500 nm long-pass filter, 1200 nm long-pass filter, 1100 nm long-pass filter, 1000 nm long-pass filter, 900 nm long-pass filter, or 850 nm band-pass filter were applied to collect the emission of the probes. Except the 1500 nm long-pass filter, other filters were used to collect the emission of the ICG. Exposure time was 100 ms for all images.

### Dynamic imaging in the NIR-IIb window

In order to visualize the vascular dynamically, the mice were intravenously injected with 100 µL probes and a series of NIR-IIb photos were gathered by a 640 × 512 pixel InGaAs-NIRvana640LN camera. For recording the heartbeat and respiration, the mice were fixed in the supine position on a console. The probes were injected into the mice and the video began with the injection. The heart beats and breath rates were calculated by changes in the intensity of the heart and lung over time. As for observing the lymphatic system in the NIR-IIb windows, we opened the belly and fixed the skin in the sides. Next, the probes (10 µL) were injected into the subiliac lymph nodes and the video of this progress was recorded. Exposure time was 100 ms for all images.

### Dynamic NIR-IIb imaging of the thrombolysis

For inducing the thrombus model successfully, we used the FeCl_3_ method according to the early literatures. First, we placed the mice as the supine position, and then, removed the skin of mice to expose the femoral artery or carotid artery. Next, we put the FeCl_3_-soaked filter paper (10 wt%) on the exposed vessels. After 5 min, we removed the filter paper and injected the probes (100 µL) into mice. Later, urokinase (100 U/mL) was injected into the mice for thrombolysis. All the NIR-IIb data were collected by the InGaAs-NIRvana640LN camera.

### Dynamic NIR-IIb imaging of the ischemic reperfusion in hindlimbs

Ischemic mouse models were established by ligation of the femoral vein and artery using hemostatic clip for different durations (40 min or 1.5 h) to generate different degrees of ischemia. The probe was then intravenously injected into hindlimb ischemia mice. Later, we removed the clips to observe the ischemic reperfusion in limbs. The dynamic and static data were gathered with the InGaAs-NIRvana640LN camera. The exposure time was 300 ms for all the data. The blood flow velocity (BFV) was calculated by the time among the three regions of interest (ROI) and the distance among the three ROI.

### NIR-IIb imaging of monitoring tumor growth

To establish the breast cancer model, approximately 5×10^5^ 4T1 cells in 25 µL PBS were injected to the right hindlimb of the BALB/c mice subcutaneously. To visualize the tumor growth, we injected the probes (75 µL) into the mice intravenously at 0, 3 and 5 days. All the NIR-IIb images were collected by InGaAs-NIRvana640LN camera and Series II 900/1700-H. For testing the biodistribution of the mice, BALB/c was injected the probes (100 µL) intravenously and imaged at different time with a 100 ms exposure time. The mice were placed as supine position and prone position, respectively.

## Results and Discussion

### Synthesis and characterization of the Er^3+^/Ce^3+^ co-doped core/shell nanoparticles with enhanced DC emission

**Figure 1:**
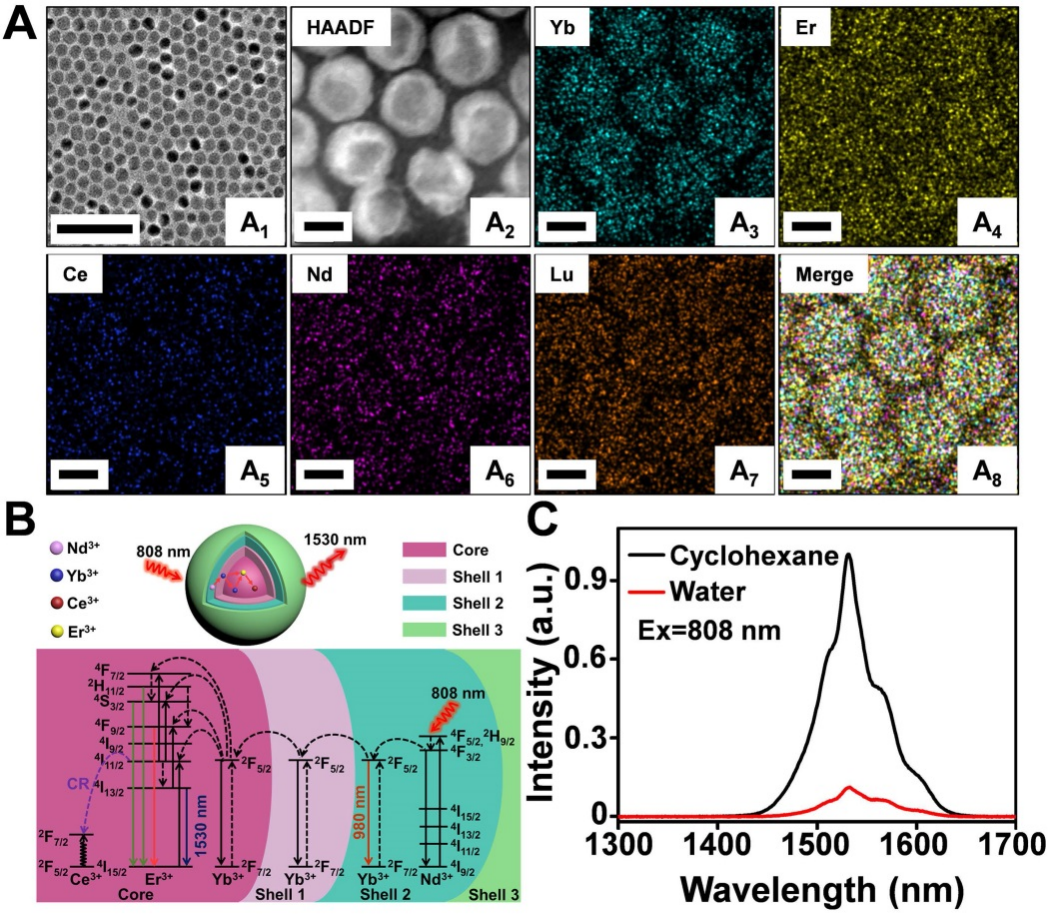
** (A)** Representative TEM image (A_1_), HAADF-STEM image (A_2_) and corresponding element mapping image of Er^3+^/Ce^3+^ co-doped nanoparticles, Yb (A_3_), Er (A_4_), Ce (A_5_), Nd (A_6_), Lu (A_7_) and Merge (A_8_). Scale bar: 100 nm (A_1_), Scale bar: 10 nm (A_2_-A_8_). **(B)** Luminescence mechanisms involved in the generation of the intense 1530 nm emission in Er^3+^/Ce^3+^ co-doped nanoparticles excited at 808 nm. **(C)** The NIR IIb emission spectra of Er^3+^/Ce^3+^ co-doped-RENPs dispersed in cyclohexane and Er-DCNPs dispersed in water under 808 nm light excitation.

A modified layer-by-layer high-temperature co-precipitation method was used to synthesize a series of nanoparticles with varied interlayers and dopant concentration. Finally, the composition of nanoparticles was optimized to be NaYbF_4_:2%Er,2%Ce@NaYbF_4_@NaNdF_4_:50%Yb@NaLuF_4_ with a core/shell1/shell2/shell3 (C/S1/S2/S3) structure (Figure S1A-D and Figure S2A) based on corresponding optical properties (Figure S2B-F). A representative TEM image of NaYbF_4_:2%Er,2%Ce@NaYbF_4_@NaNdF_4_:50%Yb@NaLuF_4_ revealed uniform and monodispersed spherical particles with a diameter of 16.2 ± 1.7 nm (Figure 1A_1_). High-angle annular dark field-scanning TEM (HAADF-STEM) (Figure 1A_2_) and corresponding elemental mapping analysis of Er^3+^/Ce^3+^ co-doped nanoparticles (Figure 1A_3_-A_8_) confirmed the presence of Yb (green), Er (yellow), Ce (blue), Nd (pink), and Lu (orange) in the C/S1/S2/S3 nanoparticles. The energy transfer process of the Nd^3+^-Yb^3+^-Er^3+^-Ce^3+^ system is presented in Figure 1B. After light excitation at 808 nm, the Nd^3+^ ions in Shell 2 served as the sensitizer to reach the excited state of the ^4^F_5/2_ state of Nd^3+^ and transfer its energy to the adjacent Yb^3+^ ions, resulting in a population of the ^2^F_5/2_ state of Yb^3+^. The excited ^2^F_5/2_ state of Yb^3+^ then radiatively relaxed to its ^2^F_7/2_ energy level, emitting NIR luminescence at 980 nm. Some of the excitation energy migrated over the Yb^3+^ sublattice and was finally trapped by the activator ions of Er^3+^ in the core. Subsequently, the excited ^4^I_11/2_ state of Er^3+^ nonradiatively decayed into the ^4^I_13/2_ level and allowed NIR-IIb DC emission with a maximum at 1530 nm. However, the ^4^I_11/2_ state of Er^3+^ could also be excited to the higher ^2^H_11/2_ and ^4^S_3/2_ levels by further absorbing the excitation energy from Nd^3+^ to generate UC luminescence, competing with the DC process. Therefore, we doped the Ce^3+^ ions into the core of the particle to boost the DC pathway while suppressing the UC process, as the interionic cross-relaxation (CR) between Er^3+^ (^4^I_11/2_-^4^I_13/2_) and Ce^3+^ (^2^F_5/2_-^2^F_7/2_) accelerated the nonradiative relaxation of Er^3+^ from ^4^I_11/2_ to ^4^I_13/2_ 40-44. Accordingly, the DC luminescence of Er^3+^/Ce^3+^ co-doped nanoparticles at 1530 nm was dramatically increased by Ce^3+^ doping (Figure 1C). The absolute quantum yield of NaYbF_4_:2%Er,2%Ce@NaYbF_4_@NaNdF_4_:50%Yb@NaLuF_4_ nanoparticles in cyclohexane was determined to be 35.6% under the laser excitation of 100 mW/cm^2^.

For *in vivo* biomedical imaging, the hydrophobic NaYbF_4_:2%Er,2%Ce@NaYbF_4_@NaNdF_4_:50%Yb@NaLuF_4_ nanoparticles were further modified with mPEG5000 to produce a hydrophilic surface (named Er-DCNPs) 40. As illustrated in Figure S3A-B, the hydrodynamic diameter of nanoparticels gradually increased and its surface charge shifted to more positive values, demonstrating the successful surface modification. As shown in Figure S3C, the hydrodynamic size of the hydrophilic nanoparticles remained nearly unchanged over 24 h in water, phosphate buffer solution (PBS), and medium solutions, respectively, indicating its excellent stability and dispersibility in the physiological environment. Moreover, Er-DCNPs showed great photostability under 808 nm laser irradiation (Figure S3D-E).

### Evaluation of NIR-IIb fluorescence imaging performance of Er-DCNPs in tissue phantoms

**Figure 2:**
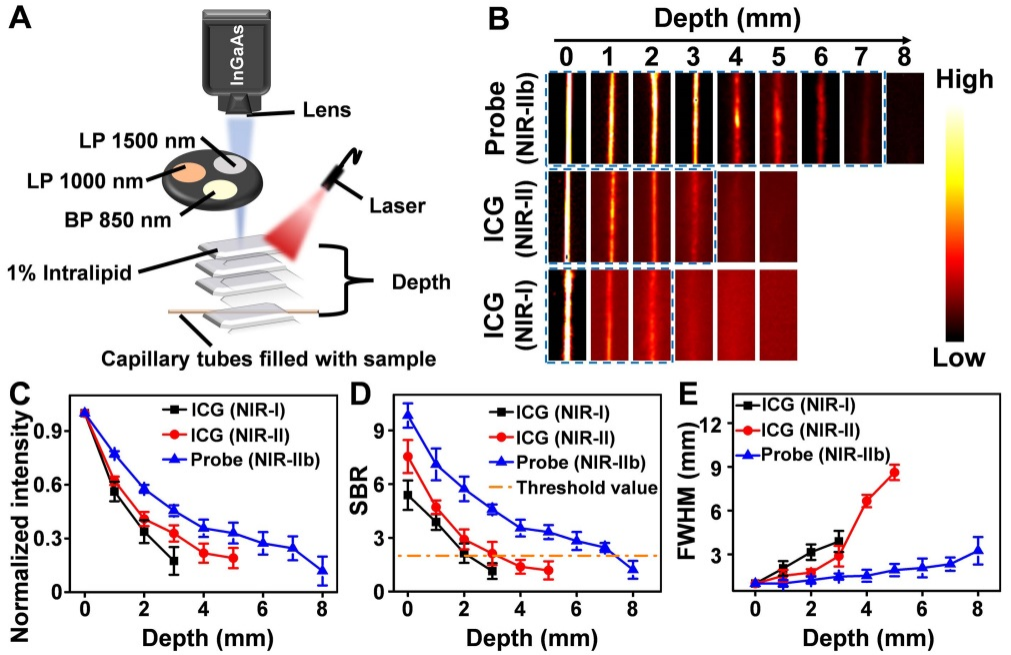
** (A)** A home-built fluorescence imaging setup for the tissue phantom study. **(B)** Fluorescence images of capillaries filled with the Er-DCNPs and ICG, immersed in 1% Intralipid at varying depths. Fluorescent signals of Er-DCNPs and ICG were collected using 1500 nm, 1000 nm long-pass filters, or an 850 nm band-pass filter. **(C)** The normalized intensity of the capillary images is shown in (B). **(D)** The SBR of the capillary images is shown in (B). **(E)** The full width at half maximum (FWHM) for the capillaries is shown in (B). The bars represent mean ± standard deviation derived from n = 3 lines tested at varying positions in the capillary images.

As mentioned above, fluorescence imaging in the NIR-IIb window benefits from its further reduced light scattering and tissue autofluorescence, which was expected to cause higher spatial resolution and deeper tissue penetration. Thus, before *in vivo* imaging, a tissue phantom study using Intralipid that mimics the optical properties of biological tissues was performed to compare the imaging performance of Er-DCNPs with that of indocyanine green (ICG), a clinically approved dye with NIR-I and NIR-II emission (Figure 2A). The capillary tubes filled with Er-DCNPs or ICG solutions were immersed in a 1% Intralipid solution at an increased phantom depth. To optimize the signal collection conditions, a 1500 nm long-pass filter (LP 1500), 1000 nm long-pass filter (LP 1000), or 850 nm band-pass filter (BP 850) was selected to acquire NIR-IIb imaging for Er-DCNPs and NIR-II or NIR-I imaging for ICG using a home-built InGaAs setup (Figure 2A). The Gaussian-fitted full width at half maximum (FWHM) and SBR were used to evaluate the resolution and clarity of the images. As shown in Figure 2B and C, with an increase in penetration depth from 0 to 3 mm, the images of capillary tubes taken in the NIR-I and NIR-II windows became blurry and even invisible 45. In contrast, the NIR-IIb imaging results of Er-DCNPs could resolve sharp edges of the capillary at a depth of up to 7 mm with the SBR passing the threshold value of 2 (Figure 2D), which was defined as the minimum value required to resolve the capillary profile *in vitro* 46, suggesting a higher imaging resolution and contrast achieved by Er-DCNPs at longer emission wavelengths. This result was further supported by the FWHM measurements of capillary images at varying depths, which clearly showed that Er-DCNPs enabled better feature integrity than ICG imaging at the same position due to the low scattering and autofluorescence interference at 1530 nm (Figure 2E), ultimately indicating the great potential of Er-DCNPs for deep tissue imaging *in vivo*.

### *In vitro* and *in vivo* biocompatibility of Er-DCNPs

The cytotoxicity of different concentrations of Er-DCNPs (0-500 μg/mL) against 4T1 cells was evaluated using the 3-(4,5-dimethyl-2-thiazolyl)-2,5-diphenyl-2-H-tetrazolium bromide (MTT) assay. As shown in Figure S4A, the viability of 4T1 cells remained over 80% after 24 h of incubation, even at a high Er-DCNPs concentration (500 μg/mL), indicating the low cytotoxicity of Er-DCNPs. To test the *in vivo* toxicity of Er-DCNPs, we performed a 7-day post-injection study. No abnormal behaviors or obvious body weight drop was observed in mice administered Er-DCNPs during the entire observation period (Figure S4B). Meanwhile, the blood biochemistry analysis of treated mice showed that all parameters were maintained within the normal range (Figure S5), revealing negligible side effects in mice. Additionally, the pharmacokinetic behavior of Er-DCNPs *in vivo* was evaluated after intravenous injection. As shown in Figure S6, the fluorescence signals gradually decreased after 2 h, and were mainly observed in the liver and spleen. These results suggest that Er-DCNPs can be safely utilized as excellent NIR-IIb fluorescence probe for *in vivo* bioimaging.

### High-resolution NIR-IIb fluorescence imaging of vasculature with Er-DCNPs

Encouraged by the superior *in vitro* imaging performance and good biocompatibility of Er-DCNPs, we further explored the feasibility of applying Er-DCNPs for vasculature imaging *in vivo*. Acquiring accurate and detailed information on normal vascular anatomy is a prerequisite for real-time monitoring of dynamic changes in blood flow caused by PAD. Therefore, the blood vessels of the brain, hindlimb, and lymphatic system in healthy mice were detected with Er-DCNPs to assess the spatial resolution of Er-DCNPs for *in vivo* vascular imaging. ICG was used as a control.

For regional cerebral vasculature imaging in the mouse brain, C57BL/6 mice were intravenously injected with Er-DCNPs or ICG in PBS solution at the same dose. Different wavelength filters were selected to obtain optimal fluorescence images for Er-DCNPs and ICG at 1 min post-injection. As shown in Figure S7A, as the imaging window shifted toward longer wavelengths, the cerebral vessels were gradually illuminated with enhanced sharpness for both groups, mainly because of reduced light attenuation at longer wavelength. However, compared with smeared fluorescence images of mouse brains from the NIR-I and NIR-II windows with ICG, Er-DCNPs offered a markedly clearer delineation of cerebrovascular structures in the NIR-IIb window with suppressed background. Small cortical vessels could also be clearly identified, indicating the excellent spatial resolution of vessel imaging enabled by the Er-DCNPs. The cross-sectional fluorescence profile from selected vessels in the brain revealed a large difference in the FWHM of the fluorescence signals originating from Er-DCNPs and ICG (Figure S7B). Moreover, when the same filter (LP 1000 nm) was employed, the Er-DCNPs produced a sharper FWHM than that of ICG (115 µm *vs.* 282 µm). In particular, the cerebral vessel, which was as small as 63 μm, could be discerned beyond 1500 nm with Er-DCNPs administration, demonstrating the superior vessel imaging capacity of Er-DCNPs in the NIR-IIb region. Consistent results were obtained for NIR-IIb imaging of blood vessels in the hindlimb and back of mice after Er-DCNPs injection (Figure S8A-B), where the complex vascular network with many tiny capillary branches was finely delineated by NIR-IIb fluorescence signals from Er-DCNPs.

**Figure 3:**
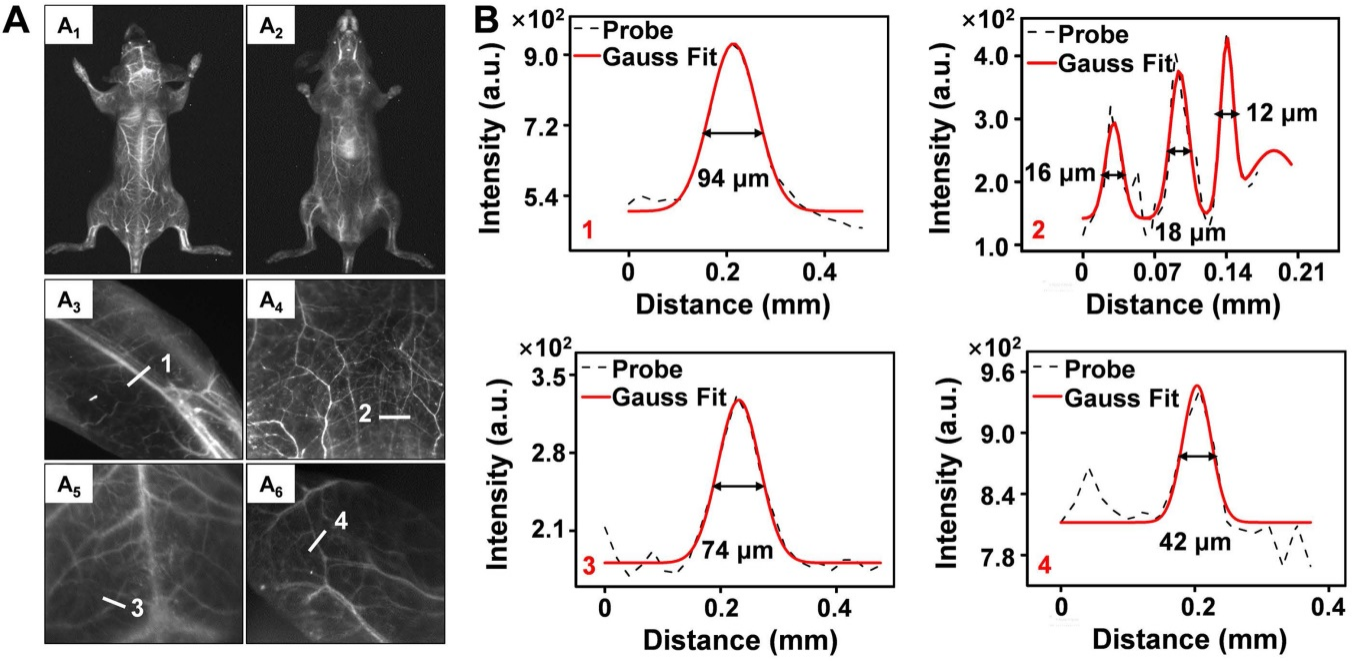
** (A)** NIR-IIb bioimaging of mice whole-body (A_1_-A_2_), hindlimb (A_3_), abdomen (A_4_), brain (A_5_) and ear (A_6_) after intravenous injection of Er-DCNPs (n = 3). **(B)** The fluorescence intensity profiles (dashed black line) and Gaussian fitting curve (solid red line) along white lines 1-4 in the NIR-IIb fluorescence image are shown in (A).

To further evaluate the performance of Er-DCNPs for deep tissue imaging, whole-body vascular imaging was conducted with normal mice in the supine and prone positions. As shown in Figure 3A_1_-A_2_, the whole-body vasculature could be distinguished from the background upon intravenous injection of Er-DCNPs under 808 nm excitation (1500 nm long-pass filter). Benefiting from the exceptional spatial resolution and improved penetration depth of Er-DCNPs, more detailed information about the blood vessels can be acquired from high-magnification images of the hindlimb, abdomen, brain, and ear (Figure 3A_3_-A_6_ and Figure 3B), demonstrating the great potential of Er-DCNPs for fluorescence angiography *in vivo*. Such outstanding bioimaging performance of Er-DCNPs in the NIR-IIb window also enabled the visualization of elaborate vasculature in the intestine and kidney with sharp FWHM (Figure S9A-E).

### High-resolution NIR-IIb fluorescence imaging for tumor angiogenesis

In contrast to normal vasculature, tumor blood vessels are functionally abnormal, which facilitates tumor formation, progression, and metastasis. Therefore, accurate evaluation of tumor-associated blood vessels and microvascular networks could provide valuable insights into tumor growth 47,48. For tumor vascular imaging, NIR-IIb images were acquired after 1-min injection of Er-DCNPs into the breast tumor-bearing BALB/c mice on days 3 and 5, respectively. As the tumor grew, new blood vessels were observed with intensified fluorescence signals (Figure S10A). Moreover, accompanied by an increase in major blood vessels, abundant small capillary networks gradually appeared. As depicted in Figure S10B-C, the density and diameter of the blood vessels markedly increased throughout tumor development, showing the great spatiotemporal resolution of Er-DCNPs that can be utilized to identify tumor-associated vasculatures.

### Real-time monitoring of hemodynamic parameters using NIR-IIb fluorescence imaging

**Figure 4:**
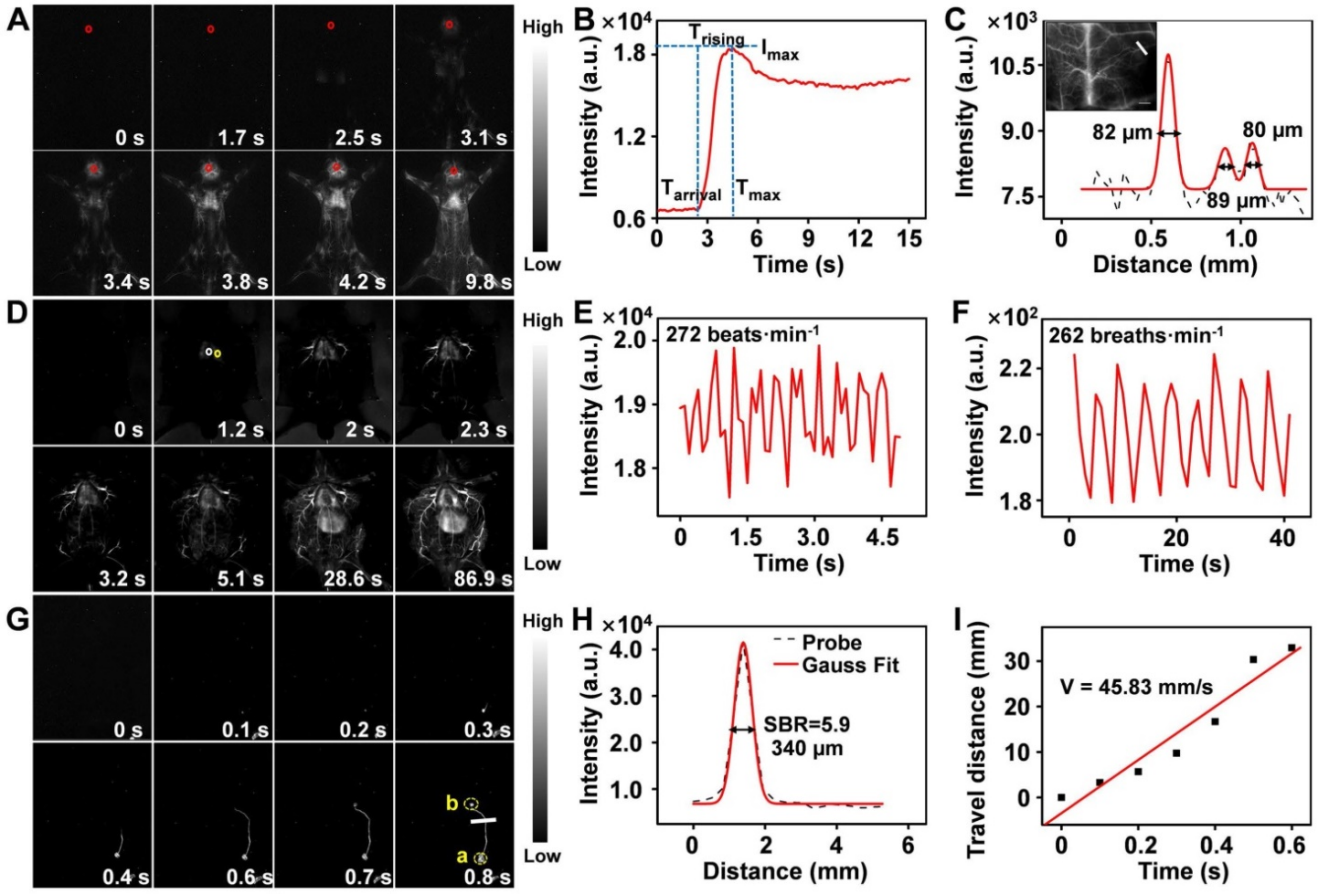
** (A)** Real-time NIR-IIb fluorescence images of mouse back vessels. **(B)** Relative fluorescence intensity of the regions of interest (ROI, red circle in the brain) over time of injected Er-DCNPs. The arrival time (T_arrival_) of Er-DCNPs and the time of the maximum intensity (T_max_) are indicated on the time axis. The rising time (T_rising_) was calculated as T_max_ - T_arrival_. **(C)** The fluorescence intensity profiles (dashed black line) and Gaussian fitting curve (solid red line) along the white line in the NIR-IIb fluorescence image are shown in the inset. The inset shows NIR-II bioimaging of mice brain after intravenous injection of Er-DCNPs. **(D)** Real-time NIR-IIb fluorescence images of an *in situ* dissected mouse after injection of Er-DCNPs. White circle indicates the ROI in the heart and yellow circle indicates the ROI in the lung. **(E)** Heartbeat of the mouse in (D); the heartbeat curves recorded by the intensity of ROI in the heart. **(F)** The respiratory rate of the mouse in (D); the vibration curves recorded by the intensity of ROI in the lung. **(G)** Real-time NIR-IIb fluorescence of lymphatic vessel after injecting Er-DCNPs at popliteal lymph nodes. **(H)** The fluorescence intensity profiles (dashed black line) and Gaussian fitting curve (solid red line) along the white line in the NIR-IIb fluorescence image are shown in (G). **(I)** The lymph flow rate from a to b in (G). The slope of the function was calculated as the lymph flow rate.

Encouraged by the superior static imaging capability of Er-DCNPs, we further explored its feasibility for real-time monitoring of the vascular changes, which could provide both anatomic and hemodynamic information to facilitate a quick and reliable identification of PAD as well as an accurate assessment of the therapeutic effects. Er-DCNPs solution was injected into a normal mouse, and whole-body fluorescence imaging was carried out at a resolution of 640 × 512 pixels. As shown in Figure 4A and Supplementary Movie 1, back vessels were distinctly visualized at 3.4 s after injection. Further, even small vessels with different diameters could be identified with high spatial resolution (Figure S11A and B) owing to the minimal tissue scattering and auto-fluorescence of NIR-IIb emission. Meanwhile, the injection site was gradually lit due to leakage of the tail-vein injected probe, indicating the great potential of the probe for intraoperative anastomosis assessment (Figure S12). Based on Supplementary Movie 1, NIR-IIb fluorescence signals in the brain blood vessels of the mouse were measured in the first 20 s immediately after intravenous injection of Er-DCNPs. As shown in Figure 4B, intense fluorescence could be detected at 2.4 s (as T_arrival_) post-injection of Er-DCNPs and could gradually reach the maximum (as I_max_) at 4.5 s (as T_max_). The intensity of signals stabilized after 7.1 s injection. T_rising_ was obtained by subtracting T_arrival_ from T_max_ to represent the status of the tissue blood supply, suggesting that Er-DCNPs could monitor hemodynamic changes *in vivo*. In addition, the cerebral vasculature imaging results in Figure 4C reconfirmed the superior imaging ability of Er-DCNPs.

Furthermore, we performed a time series fluorescence imaging of an *in situ* dissected mouse. As Er-DCNPs circulated from larger vessels into smaller vessels, fluorescence signals from tiny vasculatures gradually intensified and reached a maximum at 86.9 s post-injection. The video (Supplementary Movie 2) showed that Er-DCNPs reached the heart first and then entered the pulmonary circulation before being pumped into the whole body. Owing to reduced light scattering and minimal autofluorescence interference in the NIR-IIb window, an extensive branched network of blood vessels was well outlined throughout the body with excellent spatial resolution (Figure 4D). The high-magnification image of blood vessels in Figure 4D clearly shows that our Er-DCNPs were preferentially distributed in the larger vessels and then throughout the smaller vessels. The SBR value and the blood flow velocity (BFV) of ROI could be easily calculated, facilitating a thorough understanding of blood flow features and their interaction with the adjacent vessels *in vivo* (Figure S13A-C).

In addition to blood vessels, the profiles of deeper organs, such as the heart, lung, and liver can be precisely delineated by Er-DCNPs, enabling real-time, contact-free monitoring of critical physiological parameters, such as heart and respiratory rate. The heart rate of the healthy mouse was 272 beats/min (Figure 4E), which was comparable to that of a previous report 49. Meanwhile, the respiratory rate of the mouse was 262 breaths/min (Figure 4F) by extracting the fluorescence signals from a series of images of lung vibration 50. Superb temporal and spatial resolution offered by Er-DCNPs were also well reflected in lymphatic vessel imaging *in vivo* (Figure 4G and Supplementary Movie 3), where an SBR of 5.9 was obtained, surpassing the Rose criterion (SBR 5), where lymphatic vessels can be resolved with high certainty (Figure 4H) 51. We further tracked the flow of lymph fluid from the subiliac lymph nodes to the accessory axillary lymph nodes using Er-DCNPs, which yielded a fast lymph flow rate of 45.83 mm/s (Figure 4I). This result indicated that Er-DCNPs could be utilized for accurate visualization of lymphatic drainage, which is essential for evaluating the status of lymph nodes and tracing tumor metastasis via lymphatic vessels. These results indicate that Er-DCNPs could be very effective for real-time imaging of vascular structures and blood vessel-associated dynamic changes *in vivo* with higher temporal and spatial resolution.

### Real-time monitoring of the thrombolysis processes in a mouse model

**Figure 5:**
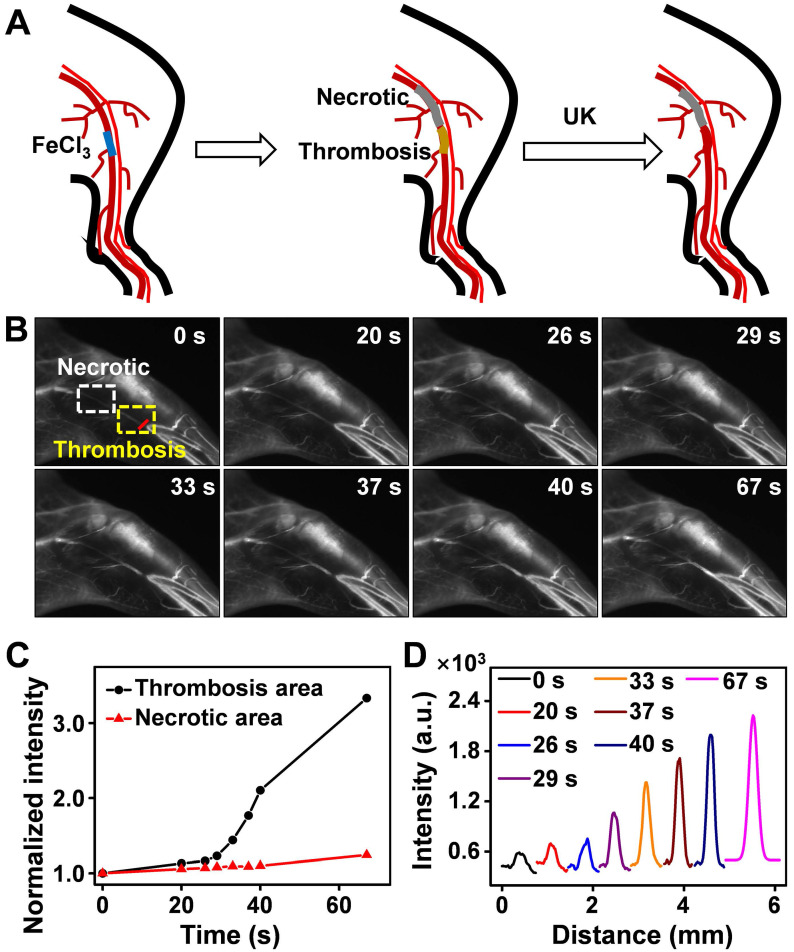
** (A)** Schematic illustration of the thrombolysis process at the femoral artery. **(B)** Real-time NIR-IIb imaging of femoral artery by Er-DCNPs at different time points of urokinase (UK) injection. Thrombotic and necrotic areas were respectively marked in yellow and white (n = 3). **(C)** Changes in the intensity of the thrombotic and necrotic areas in (B) over time. **(D)** Thrombotic plaque decreased in size over time. Plaque size was calculated based on signals of red line marked in (B).

**Figure 6:**
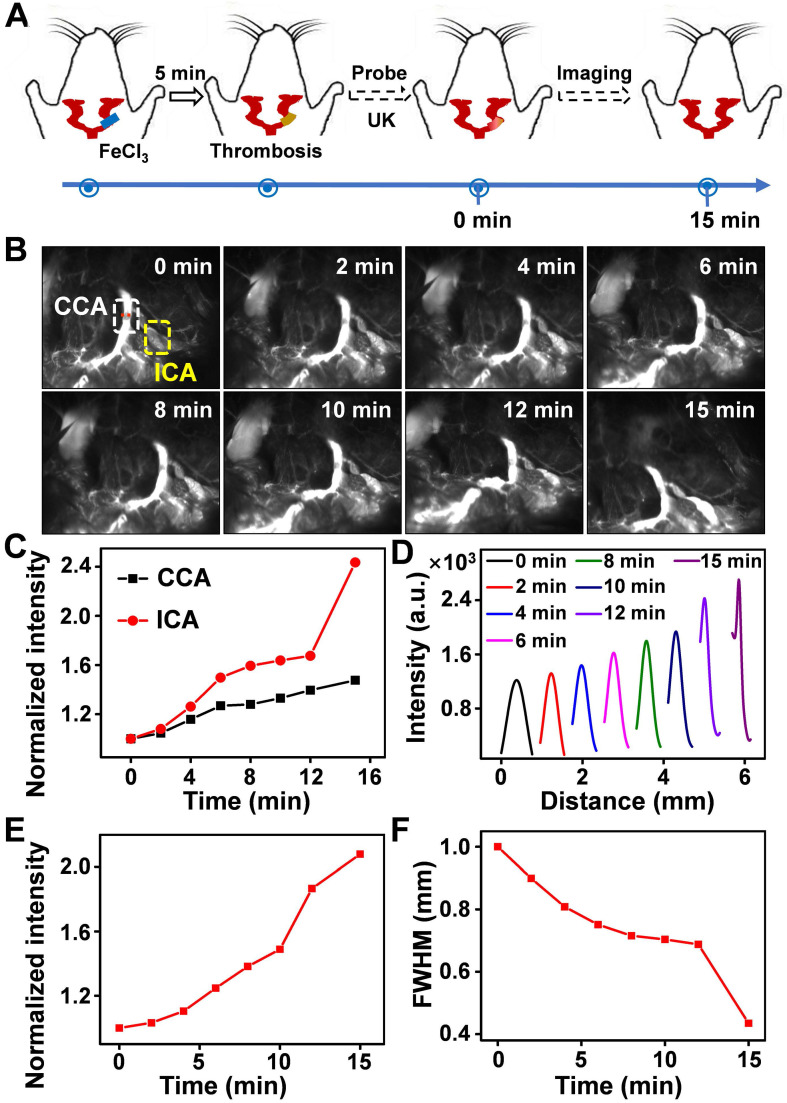
** (A)** Schematic illustration of the thrombolysis process at the carotid artery. **(B)** Real-time NIR-IIb imaging of CCA (white area) and ICA (yellow area) using Er-DCNPs at different time points of UK injection (n = 3). **(C)** Changes in the intensity of CCA and ICA in (B) at varying time points. **(D)** Thrombotic plaque of CCA decreased in size. Plaque size was calculated based on signals of dashed red line marked in (B). **(E-F)** Intensity and FWHM of the thrombotic plaque at varying time points, which were calculated from (D).

Thrombus is a blood clot that forms in blood vessels with high rates of acute mortality and long-term disability. Timely diagnosis and evaluation of the treatment efficacy of thrombosis are crucial for reducing morbidity and potential mortality [52‒55]. At present, urokinase (UK), a thrombolytic agent, has been widely used to treat thrombus in clinical practice [56‒59]. To evaluate the thrombolytic effect of UK clinically, however, four coagulation indicators must be monitored before and after its administration, which is tedious and exhausting. Moreover, an additional 2 h may be needed to obtain the results, thereby failing to provide fast feedback on treatment effectiveness. As Er-DCNPs exhibited remarkable capability for real-time detection of hemodynamic changes, we were inspired to validate the *in vivo* efficacy of Er-DCNPs for evaluating UK-facilitated thrombolysis. Common femoral artery (CFA) thrombosis was induced by the FeCl3 method, and the angionecrosis model was used for comparison (Figure 5A) 60. Then Er-DCNPs or UK was intravenously injected into the CFA thrombotic mouse to observe the affected side. As shown in Figure 5B, blood vessels in the common femoral artery region can be clearly observed with high fidelity after injecting Er-DCNPs, whereas blood vessels in the affected area were invisible due to thrombosis and necrosis. Then, UK was injected into the mouse and dynamic fluorescence imaging of the thrombotic and necrotic areas of mice was performed to monitor the thrombolysis processes with a NIR-II InGaAs camera (Supplementary Movie 4). The video showed that blood vessels in the thrombotic area were gradually lit and the FWHM of fluorescence signals in this area significantly reduced with time, illustrating effective blood flow recovery due to fibrin degradation in blood clots caused by UK (Figure 5C-D and Figure S14A-D). This finding indicates that Er-DCNPs performed competently in evaluating the degree of thrombus and the efficacy of thrombolysis in real-time. In addition, we tracked thrombolysis and blood flow perfusion processes in the common carotid artery (CCA) thrombotic model using Er-DCNPs (Figure 6A). Interestingly, the thrombotic region was located exactly at the junction of the CCA and internal carotid artery (ICA), causing weak ICA signals due to the occlusion of the vasculature (0 min in Figure 6B). As shown in Figure 6B-C, after intravenous administration of UK, the fluorescence intensity of ICA increased by nearly 2.8-fold within 15 min, suggesting an effective blood recovery. Meanwhile, NIR-IIb signals in the thrombotic plaque were enhanced by 2.2-fold and FWHM significantly reduced over time (Figure 6D-F), indicating that the thrombotic plaque reduces in size before disappearing. Altogether, Er-DCNPs markedly benefited from their higher temporal and spatial resolution and could be successfully used for the rapid and accurate imaging of vascular-related diseases and real-time evaluation of therapeutic effectiveness.

### Real-time monitoring of the reperfusion processes of mouse in a PAD model

**Figure 7:**
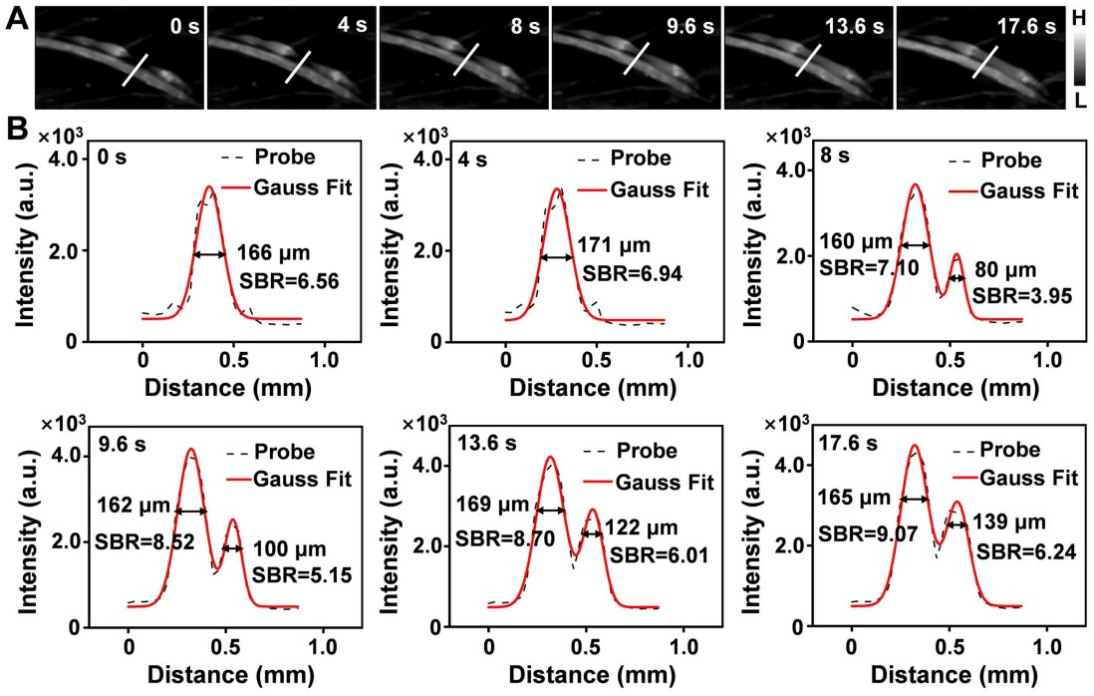
** (A)** Real-time NIR-IIb imaging of the ischemic perfusion in the femoral vein of the affected region (upper) and the normal region (lower) (n = 3). **(B)** Fluorescence intensity profiles (dashed black line) and Gaussian fitting curve (solid red line) along the white line in the NIR-IIb fluorescence image are shown in (A).

Limb ischemia is a common form of PAD. The dynamic assessment of the degree of ischemia and monitoring of vascular reperfusion are important for the diagnosis and treatment of PAD 16,61,62; however, current clinical detection techniques cannot perform these tasks. To determine whether Er-DCNPs with 1530 nm emission could be adequate for the imaging of ischemic hindlimb, Er-DCNPs were intravenously injected into hindlimb ischemia mice to compare the normal and affected regions. The dynamic process of ischemic reperfusion was further monitored by NIR-II fluorescence imaging (Supplementary Movie 5). As shown in Figure 7A, NIR-IIb fluorescence imaging unambiguously resolved the normal femoral side, while fluorescence signals on the ischemic side were obviously interrupted by ligation of the femoral artery and vein, which resulted in the occlusion of blood flow. Importantly, real-time fluorescence imaging depicted the recovery of ischemic hindlimbs. Consistently, the cross-sectional fluorescent profile of representative vessels revealed changes in signal distribution (Figure 7B). As time progressed, the FWHM of the normal blood vessel remained almost unchanged, while the SBR increased gradually and all SBR values surpassed the Rose criterion to clearly distinguish vascular features from the background. In contrast, ischemic blood vessels showed increased FWHM and SBR. The SBR did not surpass the Rose criterion until the surgical suture thread was removed 9.6 s later. By measuring the FWHM of the cross-sectional signal profiles of the features, the blood width of the ischemic blood vessel was found to expand from 0 μm to 139 μm within 17.6 s, suggesting that ischemic blood vessels were gradually normalized. To further examine the utility of continuous monitoring of ischemic reperfusion in limbs with Er-DCNPs, mouse models with different degrees of hindlimb ischemia were established using hemostatic clips with various clipping durations. The probe was then injected intravenously into mice with hindlimb ischemia. For the 40-min clipping group, enhanced fluorescence signals could be quickly detected within 2 s after clip removal, indicating an almost complete reperfusion under mild ischemic conditions (Figure S15A and Supplementary Movie 6). However, when the clipping time was extended to 1.5 h, markedly delayed perfusion signals were observed in the ischemic hindlimbs of mice (Figure S15B and Supplementary Movie 7). After removal of the clip, the fluorescence signals from the affected blood vessels did not appear until 8 min later, which indicated that there might be incomplete reperfusion of the blood vessels in the mouse hindlimb. Furthermore, we selected four ROIs, called a, b, c, and d, to track the ischemic reperfusion process by dynamic NIR-IIb imaging enabled by Er-DCNPs (Figure S15B). After removing the clip, the BFV of the ROIs were calculated to be 0.51 mm/s, 0.065 mm/s, and 0.026 mm/s, respectively (Figure S15C). Accordingly, the average diameters of blood vessels (for a→b, b→c, c→d) were determined as 180.5 μm, 211.2 μm and 256.0 μm, respectively (Figure S16-S18 and Table S1), which is consistent with the fact that blood flow velocity is inversely proportional to the cross-sectional area of the blood vessels 63,64. However, tiny capillaries around the ROIs failed to reperfuse. All these results indicate the superior capability of Er-DCNPs to dynamically monitor vascular perfusion recovery in the PAD model, making it highly favorable for evaluating the efficacy during PAD treatment.

## Conclusions

In summary, we successfully validated the feasibility and utility of an NIR-IIb emissive probe, Er-DCNPs, for the real-time imaging of dynamic vascular structure and hemodynamic alterations related to PAD *in vivo*. Profiting from the high temporal and spatial resolution in NIR-IIb window, Er-DCNPs performed competently to provide quick and accurate information on vasculature-related physiological and pathological processes, such as thrombus and hindlimb ischemia, in a non-invasive and non-radioactive manner. Most importantly, the superior real-time imaging capability of Er-DCNPs greatly enabled a timely evaluation of treatment effectiveness by monitoring vascular recovery conditions, achieving the delicate integration of diagnostic and therapeutic imaging functions, which is particularly significant when dealing with PAD. Altogether, this study demonstrated the great potential of this multifunctional fluorescence imaging strategy for the effective detection and treatment of vasculature-related diseases.

## Supplementary Material

Supplementary figures and table.

Supplementary movie 1.

Supplementary movie 2.

Supplementary movie 3.

Supplementary movie 4.

Supplementary movie 5.

Supplementary movie 6.

Supplementary movie 7.

## Figures and Tables

**Scheme 1:**
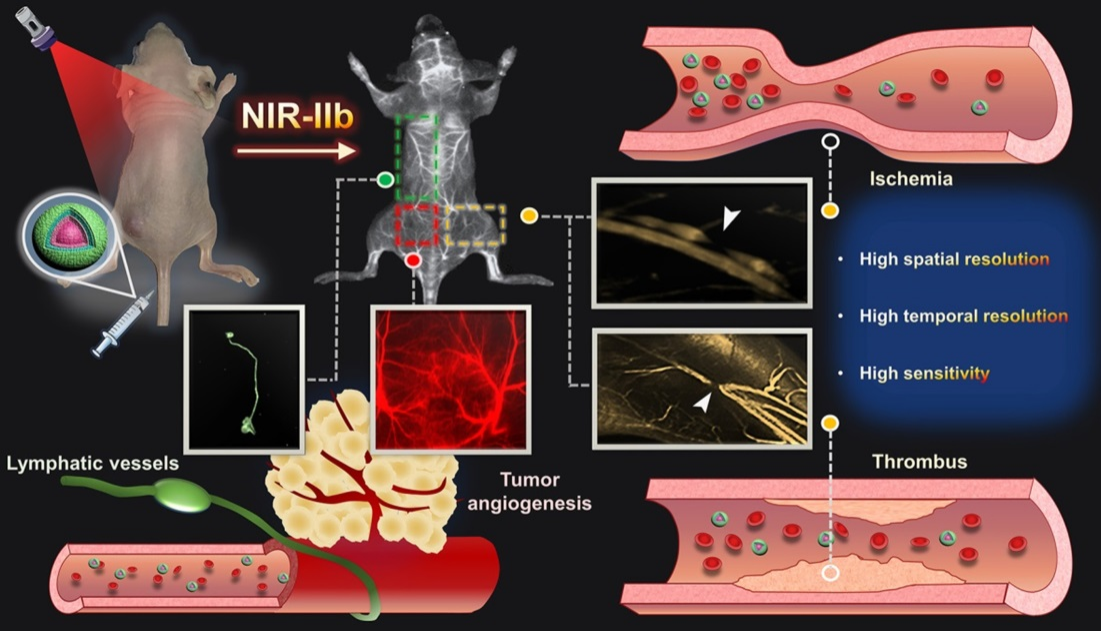
Schematic illustration of NIR-IIb probe Er-DCNPs for multifunctional biomedical imaging *in vivo* with high temporal-spatial resolution and sensitivity.

## References

[1] Criqui Aboyans (2015). Epidemiology of peripheral artery disease. Circ Res.

[2] Kullo IJ, Rooke TW (2016). Peripheral Artery Disease. N Engl J Med.

[3] Rachael L Morley, Anita Sharma, Alexander D Horsch, Robert J Hinchliffe (2018). Peripheral artery disease. BMJ.

[4] Campia U, Gerhard-Herman M, Piazza G, Goldhaber SZ (2019). Peripheral Artery Disease: Past, Present, and Future. Am J Med.

[5] Kochar A, Mulder H, Rockhold FW, Baumgartner I, Berger JS, Blomster JI, Fowkes FGR, Katona BG, Lopes RD, Al-Khalidi HR, Mahaffey KW, Norgren L, Hiatt WR, Patel MR, Jones WS (2020). Cause of Death Among Patients With Peripheral Artery Disease: Insights From the EUCLID Trial. Circ Cardiovasc Qual Outcomes.

[6] Fowkes FGR, Aboyans V, Fowkes FJI, McDermott MM, Sampson UKA, Criqui MH (2017). Peripheral artery disease: epidemiology and global perspectives. Nat Rev Cardiol.

[7] Jones DW, Farber A (2020). Review of the Global Vascular Guidelines on the Management of Chronic Limb-Threatening Ischemia. JAMA Surg.

[8] Fowkes FGR, Rudan D, Rudan I, Aboyans V, Denenberg JO, McDermott MM, Norman PE, Sampson UKA, Williams LJ, Mensah GA, Criqui MH (2013). Comparison of global estimates of prevalence and risk factors for peripheral artery disease in 2000 and 2010: a systematic review and analysis. Lancet.

[9] Morooka H, Tanaka A, Inaguma D, Maruyama S (2020). Peripheral artery disease at the time of dialysis initiation and mortality: a prospective observational multicenter study. BMJ Open.

[10] Collins R (2007). Duplex ultrasonography, magnetic resonance angiography, and computed tomography angiography for diagnosis and assessment of symptomatic, lower limb peripheral arterial disease: systematic review. BMJ.

[11] Li M, Li Z, Gao P, Jin L, Li L, Zhao W, Zhang W, Sun YL, Zhao Y, Cui JS (2020). Quantitative evaluation of postintervention foot blood supply in patients with peripheral artery disease by computed tomography perfusion. J Vasc Surg.

[12] Cavallo AU, Koktzoglou I, Edelman RR, Gilkeson R, Mihai G, Shin T, Rajagopalan S (2019). Noncontrast Magnetic Resonance Angiography for the Diagnosis of Peripheral Vascular Disease. Circ Cardiovasc Imaging.

[13] Divakaran S, Sobieszczyk PS, Di Carli MF (2020). The Potential of PET in the Management of Peripheral Arterial Disease. JACC Cardiovasc Imaging.

[14] Aboyans V, Ricco JB, Bartelink MLEL, Bjorck M, Brodmann M, Cohnert T, Collet JP, Czerny M, De Carlo M, Debus S, Espinola-Klein C, Kahan T, Kownator S, Mazzolai L, Naylor AR, Roffi M, Röther J, Sprynger M, Tendera M, Tepe G, Venermo M, Vlachopoulos C, Desormais I (2018). 2017 ESC Guidelines on the Diagnosis and Treatment of Peripheral Arterial Diseases, in collaboration with the European Society for Vascular Surgery (ESVS). Eur Heart J.

[15] Hong GS, Lee JC, Jha A, Diao S, Nakayama KH, Hou LQ, Doyle TC, Robinson JT, Antaris AL, Dai HJ, Cooke JP, Huang NF (2014). Near-infrared II fluorescence for imaging hindlimb vessel regeneration with dynamic tissue perfusion measurement. Circ Cardiovasc Imaging.

[16] Kolte D, Parikh SA, Piazza G, Shishehbor MH, Beckman JA, White CJ, Jaff MR, Iribarne A, Nguyen TC, Froehlich JB, Rosenfield K, Aronow HD (2019). Vascular Teams in Peripheral Vascular Disease. J Am Coll Cardiol.

[17] Mancusi C, de Simone G, Hitij JB, Sudano I, Mahfoud F, Parati G, Kahan T, Barbato E, Pierard LA, Garbi M, Flachskampf FA, Gerdts E (2021). Management of patients with combined arterial hypertension and aortic valve stenosis: a consensus document from the Council on Hypertension and Council on Valvular Heart Disease of the European Society of Cardiology, the European Association of Cardiovascular Imaging (EACVI), and the European Association of Percutaneous Cardiovascular Interventions (EAPCI). Eur Heart J Cardiovasc Pharmacother.

[18] Hong GS, Lee JC, Robinson JT, Raaz U, Xie LM, Huang NF, Cooke JP, Dai HJ (2012). Multifunctional *in vivo* vascular imaging using near-infrared II fluorescence. Nat Med.

[19] Hu ZH, Fang C, Li B, Zhang ZY, Cao CG, Cai MS, Su S, Sun XW, Shi XJ, Li C, Zhou TJ, Zhang YX, Chi CW, He P, Xia XM, Chen Y, Gambhir SS, Cheng Z, Tian J (2020). First-in-human liver-tumour surgery guided by multispectral fluorescence imaging in the visible and near-infrared-I/II windows. Nat Biomed Eng.

[20] Fang Y, Shang JZ, Liu DK, Shi W, Li XH, Ma HM (2020). Design, Synthesis, and Application of a Small Molecular NIR-II Fluorophore with Maximal Emission beyond 1200 nm. J Am Chem Soc.

[21] Li DF, He SQ, Wu YF, Liu JQ, Liu Q, Chang BS, Zhang Q, Xiang ZH, Yuan Y, Jian C, Yu AX, Cheng Z (2019). Excretable Lanthanide Nanoparticle for Biomedical Imaging and Surgical Navigation in the Second Near-Infrared Window. Adv Sci.

[22] Liu HL, Hong GS, Luo ZT, Chen JC, Chang JL, Gong M, He H, Yang J, Yuan X, Li LL, Mu XY, Wang JY, Mi WB, Luo J, Xie JP, Zhang XD (2019). Atomic-Precision Gold Clusters for NIR-II Imaging. Adv Mater.

[23] Yang HC, Huang HY, Ma X, Zhang YJ, Yang XH, Yu MX, Sun ZQ, Li CY, Wu F, Wang QB (2021). Au-Doped Ag_2_Te Quantum Dots with Bright NIR-IIb Fluorescence for *In situ* Monitoring of Angiogenesis and Arteriogenesis in a Hindlimb Ischemic Model. Adv Mater.

[24] Zhu SJ, Yung BC, Chandra S, Niu G, Antaris AL, Chen XY (2018). Near-Infrared-II (NIR-II) Bioimaging via Off-Peak NIR-I Fluorescence Emission. Theranostics.

[25] Lei XL, Li RF, Tu DT, Shang XY, Liu Y, You WW, Sun CX, Zhang F, Chen XY (2018). Intense near-infrared-II luminescence from NaCeF_4_:Er/Yb nanoprobes for *in vitro* bioassay and *in vivo* bioimaging. Chem Sci.

[26] Liu SJ, Ou HL, Li YY, Zhang HK, Liu JK, Lu XF, Kwok RTK, Lam JWY, Ding D, Tang BZ (2020). Planar and Twisted Molecular Structure Leads to the High Brightness of Semiconducting Polymer Nanoparticles for NIR-IIa Fluorescence Imaging. J Am Chem Soc.

[27] Li Y, Liu YF, Li QQ, Zeng XD, Tian T, Zhou WY, Cui Y, Wang XK, Cheng XD, Ding QH, Wang XF, Wu JZ, Deng H, Li YQ, Meng XL, Deng ZX, Hong XC, Xiao YL (2020). Novel NIR-II organic fluorophores for bioimaging beyond 1550 nm. Chem Sci.

[28] Huang JG, Pu KY (2020). Activatable Molecular Probes for Second Near-Infrared Fluorescence, Chemiluminescence, and Photoacoustic Imaging. Angew Chem Int Ed Engl.

[29] Zhang Z, Fang XF, Liu ZH, Liu HC, Chen DD, He SQ, Zheng J, Yang B, Qin WP, Zhang XJ, Wu CF (2020). Semiconducting Polymer Dots with Dually Enhanced NIR-Iia Fluorescence for Through-Skull Mouse Brain Imaging. Angew Chem Int Ed Engl.

[30] Bashkatov AN, Genina EA, Kochubey VI, Tuchin VV (2005). Optical properties of human skin, subcutaneous and mucous tissues in the wavelength range from 400 to 2000 nm. Journal of Physics D Applied Physics.

[31] Li YB, Zeng SJ, Hao JH (2019). Non-Invasive Optical Guided Tumor Metastasis/Vessel Imaging by Using Lanthanide Nanoprobe with Enhanced Down-Shifting Emission beyond 1500 nm. ACS Nano.

[32] Wang SF, Liu L, Fan Y, El-Toni AM, Alhoshan MS, Li DD, Zhang F (2019). *In vivo* High-resolution Ratiometric Fluorescence Imaging of Inflammation Using NIR-II Nanoprobes with 1550 nm Emission. Nano Lett.

[33] Zhang MX, Yue JY, Cui R, Ma ZR, Wan H, Wang FF, Zhu SJ, Zhou Y, Kuang Y, Zhong YT, Pang DW, Dai HJ (2018). Bright quantum dots emitting at ∼1,600 nm in the NIR-IIb window for deep tissue fluorescence imaging. Proc Natl Acad Sci USA.

[34] Diao S, Blackburn JL, Hong GS, Antaris AL, Chang JL, Wu JZ, Zhang B, Cheng K, Kuo CJ, Dai HJ (2015). Fluorescence Imaging *In vivo* at Wavelengths beyond 1500 nm. Angew Chem Int Ed Engl.

[35] Li XL, Jiang MY, Zeng SJ, Liu HR (2019). Polydopamine coated multifunctional lanthanide theranostic agent for vascular malformation and tumor vessel imaging beyond 1500 nm and imaging-guided photothermal therapy. Theranostics.

[36] Sun CX, Li BH, Zhao MY, Wang SF, Lei ZH, Lu LF, Zhang HX, Feng LS, Dou CR, Yin DR, Xu HX, Cheng YS, Zhang F (2019). J-Aggregates of Cyanine Dye for NIR-II *in vivo* Dynamic Vascular Imaging beyond 1500 nm. J Am Chem Soc.

[37] Liu MH, Zhang Z, Yang YC, Chan YH (2021). Polymethine-Based Semiconducting Polymer Dots with Narrow-Band Emission and Absorption/Emission Maxima at NIR-II for Bioimaging. Angew Chem Int Ed Engl.

[38] Ma ZR, Zhang MX, Yue JY, Alcazar C, Zhong YT, Doyle TC, Dai HJ, Huang NF (2018). Near-Infrared IIb Fluorescence Imaging of Vascular Regeneration with Dynamic Tissue Perfusion Measurement and High Spatial Resolution. Adv Funct Mater.

[39] Zhong YT, Ma ZR, Zhu SJ, Yue JY, Zhang MX, Antaris AL, Yuan J, Cui R, Wan H, Zhou Y, Wang W, Huang NF, Luo J, Hu ZY, Dai HJ (2017). Boosting the down-shifting luminescence of rare-earth nanocrystals for biological imaging beyond 1500 nm. Nat Commun.

[40] Xie XJ, Gao NY, Deng RR, Sun Q, Xu QH, Liu XG (2013). Mechanistic investigation of photon upconversion in Nd(3+)-sensitized core-shell nanoparticles. J Am Chem Soc.

[41] Zhong YT, Ma ZR, Wang FF, Wang X, Yang YJ, Liu YL, Zhao X, Li JC, Du HT, Zhang MX, Cui QH, Zhu SJ, Sun QC, Wan H, Tian Y, Liu Q, Wang WZ, Garcia KC, Dai HJ (2019). *In vivo* molecular imaging for immunotherapy using ultra-bright near-infrared-IIb rare-earth nanoparticles. Nat Biotechnol.

[42] Ren F, Liu HH, Zhang H, Jiang ZL, Xia B, Genevois C, He T, Allix M, Sun Q, Li Z, Gao MY (2020). Engineering NIR-IIb fluorescence of Er-based lanthanide nanoparticles for through-skull targeted imaging and imaging-guided surgery of orthotopic glioma. Nano Today.

[43] Cao C, Wu N, Yuan W, Gu YY, Ke JM, Feng W, Li FY (2020). Ln^3+^-doped nanoparticles with enhanced NIR-II luminescence for lighting up blood vessels in mice. Nanoscale.

[44] Li YB, Jiang MY, Xue ZL, Zeng SJ (2020). 808 nm light triggered lanthanide nanoprobes with enhanced down-shifting emission beyond 1500 nm for imaging-guided resection surgery of tumor and vascular visualization. Theranostics.

[45] Wu D, Xue DW, Zhou J, Wang YF, Feng Z, Xu JJ, Lin H, Qian J, Cai XJ (2020). Extrahepatic cholangiography in near-infrared II window with the clinically approved fluorescence agent indocyanine green: a promising imaging technology for intraoperative diagnosis. Theranostics.

[46] Wang SF, Fan Y, Li DD, Sun CX, Lei ZH, Lu LF, Wang T, Zhang F (2019). Anti-quenching NIR-II molecular fluorophores for *in vivo* high-contrast imaging and pH sensing. Nat Commun.

[47] Hanahan D, Weinberg RA (2011). Hallmarks of cancer: the next generation. Cell.

[48] De Palma M, Biziato D, Petrova TV (2017). Microenvironmental regulation of tumour angiogenesis. Nat Rev Cancer.

[49] Erhardt W, Hebestedt A, Aschenbrenner G, Pichotka B, Blümel G (1984). A comparative study with various anesthetics in mice (pentobarbitone, ketamine-xylazine, carfentanyl-etomidate). Res Exp Med.

[50] Cosco ED, Spearman AL, Ramakrishnan S, Lingg JGP, Saccomano M, Pengshung M, Arús BA, Wong KCY, Glasl S, Ntziachristos V, Warmer M, McLaughlin RR, Bruns OT, Sletten EM (2020). Shortwave infrared polymethine fluorophores matched to excitation lasers enable non-invasive, multicolour *in vivo* imaging in real time. Nat Chem.

[51] Hong GS, Robinson JT, Zhang YJ, Diao S, Antaris AL, Wang QB, Dai HJ (2012). *In vivo* fluorescence imaging with Ag_2_S quantum dots in the second near-infrared region. Angew Chem Int Ed Engl.

[52] Tritschler T, Kraaijpoel N, Le Gal G, Wells PS (2018). Venous Thromboembolism: Advances in Diagnosis and Treatment. JAMA.

[53] Meissner MH, Gloviczki P, Comerota AJ, Dalsing MC, Eklof BG, Gillespie DL, Lohr JM, McLafferty RB, Murad MH, Padberg F, Pappas P, Raffetto JD, Wakefield TW (2012). Early thrombus removal strategies for acute deep venous thrombosis: clinical practice guidelines of the Society for Vascular Surgery and the American Venous Forum. J Vasc Surg.

[54] Liang HY, Hong ZZ, Li SH, Song XR, Zhang D, Chen QS, Li J, Yang HH (2021). An Activatable X-Ray Scintillating Luminescent Nanoprobe for Early Diagnosis and Progression Monitoring of Thrombosis in Live Rat. Adv Funct Mater.

[55] Sun B, Hettie KS, Zhu SJ (2021). Near-infrared Fluorophores for Thrombosis Diagnosis and Therapy. Adv Ther.

[56] Wan MM, Wang Q, Wang RL, Wu R, Li T, Fang D, Huang YY, Yu YQ, Fang LY, Wang XW, Zhang YH, Miao ZY, Zhao B, Wang FH, Mao C, Jiang Q, Xu XQ, Shi DQ (2020). Platelet-derived porous nanomotor for thrombus therapy. Sci Adv.

[57] Yang PP, Zhang K, He PP, Fan Y, Gao XJ, Gao X, Chen ZM, Hou DY, Li Y, Yi Y, Cheng DB, Zhang JP, Shi L, Zhang XZ, Wang L, Wang H (2020). A biomimetic platelet based on assembling peptides initiates artificial coagulation. Sci Adv.

[58] Kadir RRA, Bayraktutan U (2020). Urokinase Plasminogen Activator: A Potential Thrombolytic Agent for Ischaemic Stroke. Cell Mol Neurobiol.

[59] Kunamneni A, Ravuri BD, Saisha V, Ellaiah P, Prabhakhar T (2008). Urokinase-a very popular cardiovascular agent. Recent Pat Cardiovasc Drug Discov.

[60] Bonnard T, Hagemeyer CE (2015). Ferric Chloride-induced Thrombosis Mouse Model on Carotid Artery and Mesentery Vessel. J Vis Exp.

[61] Li DF, Qu CR, Liu Q, Wu YF, Hu X, Qian K, Chang BS, He SQ, Yuan Y, Li YZ, Ko T, Yu AX, Cheng Z (2019). Monitoring the Real-Time Circulatory System-Related Physiological and Pathological Processes *In vivo* Using a Multifunctional NIR-II Probe. Adv Funct Mater.

[62] Misra S, Shishehbor MH, Takahashi EA, Aronow HD, Brewster LP, Bunte MC, Kim ESH, Lindner JR, Rich K (2019). Perfusion Assessment in Critical Limb Ischemia: Principles for Understanding and the Development of Evidence and Evaluation of Devices: A Scientific Statement From the American Heart Association. Circulation.

[63] Kal A, Duman E, Sezenöz AS, Ulusoy MO, Kal Ö (2018). Evaluation of retrobulbar blood flow and choroidal thickness in patients with rheumatoid arthritis. Int Ophthalmol.

[64] Akyürek O, Berkalp B, Sayin T, Kumbasar D, Kervancioğlu C, Oral D (2003). Altered coronary flow properties in diffuse coronary artery ectasia. Am Heart J.

