# Supplementary material for: Estimating dynamic vascular perfusion based on Er-based lanthanide nanoprobes with enhanced down-conversion emission beyond 1500 nm
Source: Theranostics. 2021 Oct 11;11(20):9859–72. doi: 10.7150/thno.65771 (PMC8581431; doi:10.7150/thno.65771)
Supplement: Supplementary file 1 — Supplementary figures and table. [file thnov11p9859s1.pdf]

Supporting Information

Estimating dynamic vascular perfusion based on Er-based lanthanide nanoprobes with enhanced down-conversion emission beyond 1500 nm

Qian Jia[1,‡], Zheng Li[1,‡], Mingli Bai[1], Haohao Yan[1], Ruili Zhang[1,2]✉, Yu Ji[1], Yanbin Feng[1], Zuo Yang[1], Zhongliang Wang[1,2]✉, and Jianxiong Li[3]✉

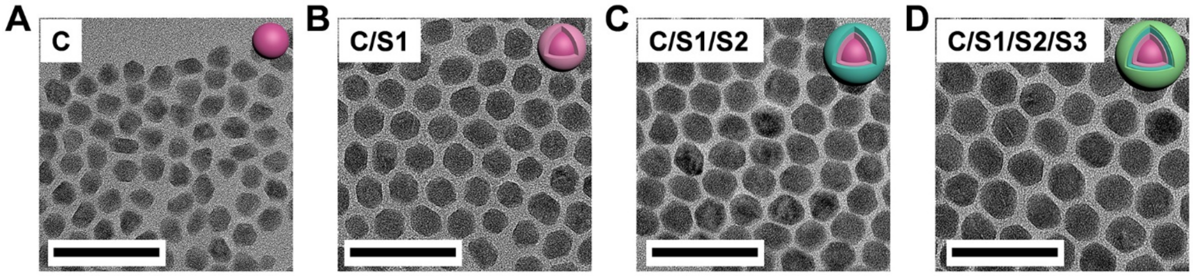

Figure S1. (A) TEM images of $NaYbF_4$:2%Er,2%Ce (Core, C). (B) TEM images of $NaYbF_4$:2%Er,2%Ce@$NaYbF_4$ (Core/Shell1, C/S1). (C) TEM images of $NaYbF_4$:2%Er,2%Ce@$NaYbF_4$@$NaNdF_4$:50%Yb (Core/Shell1/Shell2, C/S1/S2). (D) $NaYbF_4$:2%Er,2%Ce@$NaYbF_4$@$NaNdF_4$:50%Yb@$NaLuF_4$ (Core/Shell1/Shell2/shell3, C/S1/S2/S3). Scale bar: 50 nm.

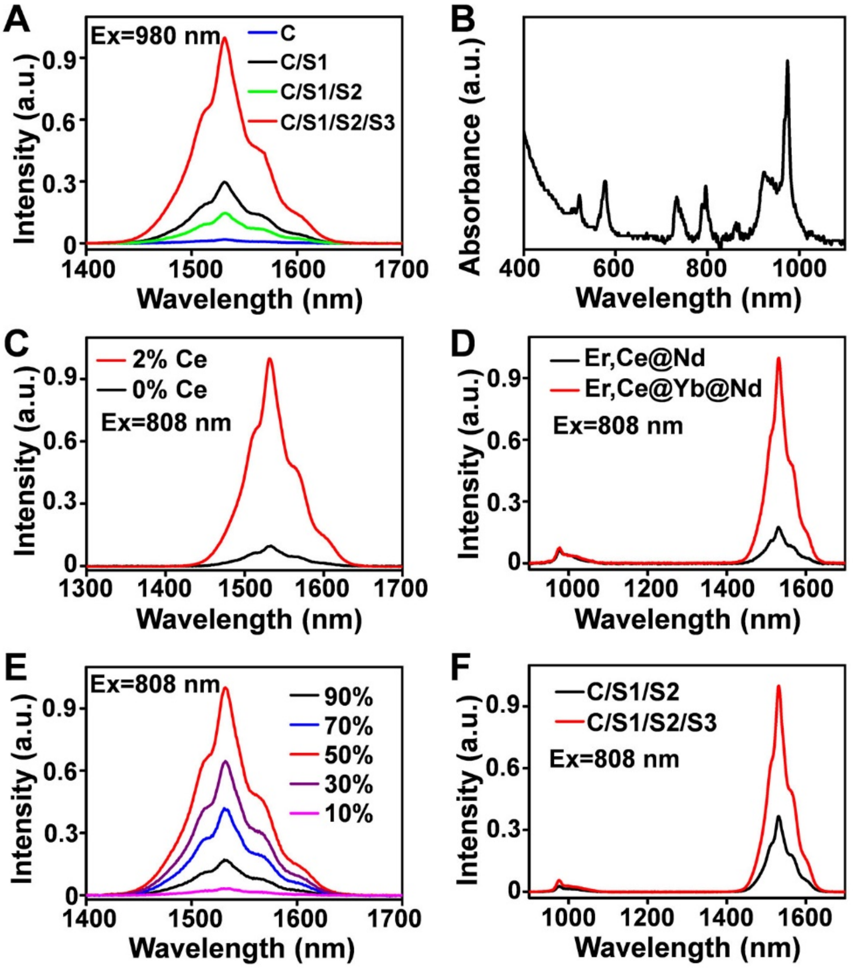

Figure S2. (A) NIR-IIb emission spectra of $NaYbF_4$:2%Er,2%Ce (C), $NaYbF_4$:2%Er,2%Ce@$NaYbF_4$ (C/S1), $NaYbF_4$:2%Er,2%Ce@$NaYbF_4$@$NaNdF_4$:50%Yb (C/S1/S2) and $NaYbF_4$:2%Er,2%Ce @$NaYbF_4$@$NaNdF_4$:50%Yb@$NaLuF_4$ (C/S1/S2/S3) under 980 laser excitation. (B) Absorbance spectra of Er-DCNPs. (C) NIR-IIb emission spectra of Er-DCNPs with different Ce^{3+} doping concentration under 808 nm laser excitation. (D) NIR-IIb emission spectra of $NaYbF_4$:2%Er,2%Ce@$NaNdF_4$:50%Yb and $NaYbF_4$:2%Er,2%Ce@$NaYbF_4$@$NaNdF_4$:50%Yb under 808 nm laser excitation. (E) NIR-IIb emission spectra of Er-DCNPs with different Yb^{3+} doping concentration under 808 nm laser excitation. (F) NIR-IIb emission spectra of $NaYbF_4$:2%Er,2%Ce@$NaYbF_4$@$NaNdF_4$:50%Yb(C/S1/S2) and $NaYbF_4$:2%Er,2%Ce@$NaYbF_4$@$NaNdF_4$:50%Yb@$NaLuF_4$ (C/S1/S2/S3) under 808 nm laser excitation.

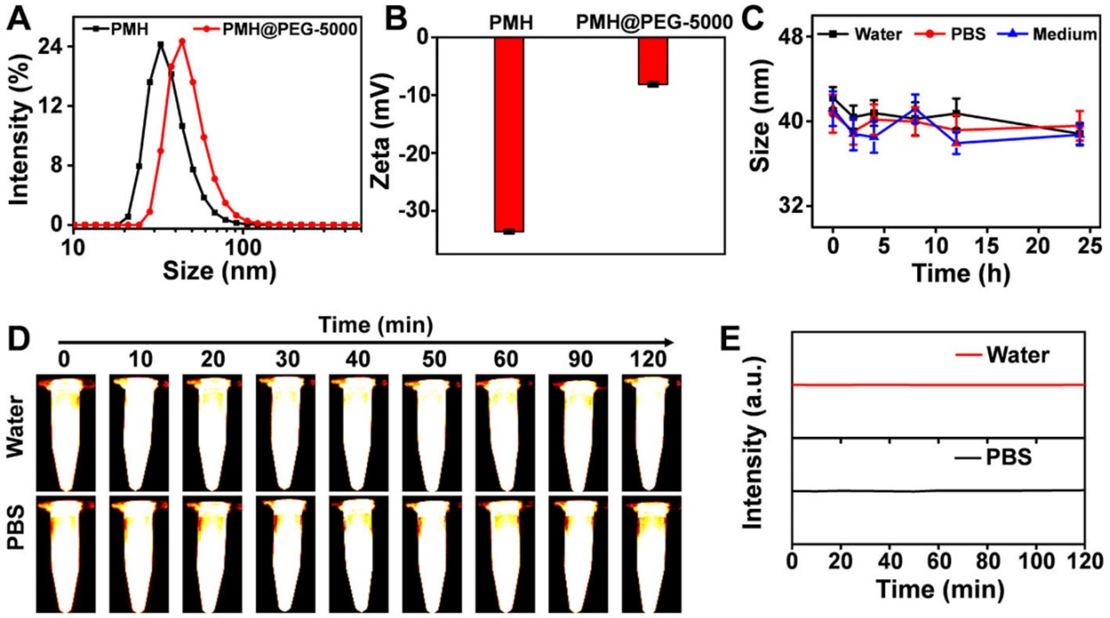

Figure S3. (A) The hydrodynamic diameter and (B) Zeta potential of PMH capped $NaYbF_4$:2%Er,2%Ce@$NaYbF_4$@$NaNdF_4$:50%Yb@$NaLuF_4$ before and after PEGylation. (C) Long-term dispersity of Er-DCNPs suspended in water, PBS and medium, respectively. (D) Photostability of Er-DCNPs in water and PBS under continuous 808 nm laser irradiation. (E) Photostability curves of Er-DCNPs in water and PBS under continuous 808 nm laser excitation (power density: 200 mW/cm^2).

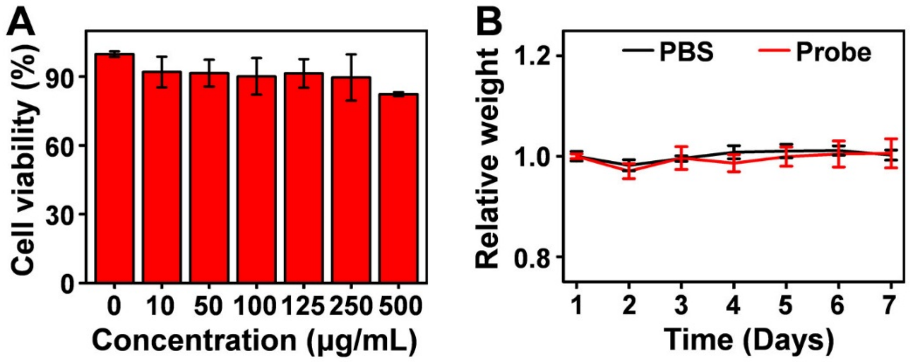

Figure S4. (A) Cell viability of 4T1 cell after treatment with different concentrations of Er-DCNPs for 24 h. (B) The weight change of mice injected with Er-DCNPs or PBS.

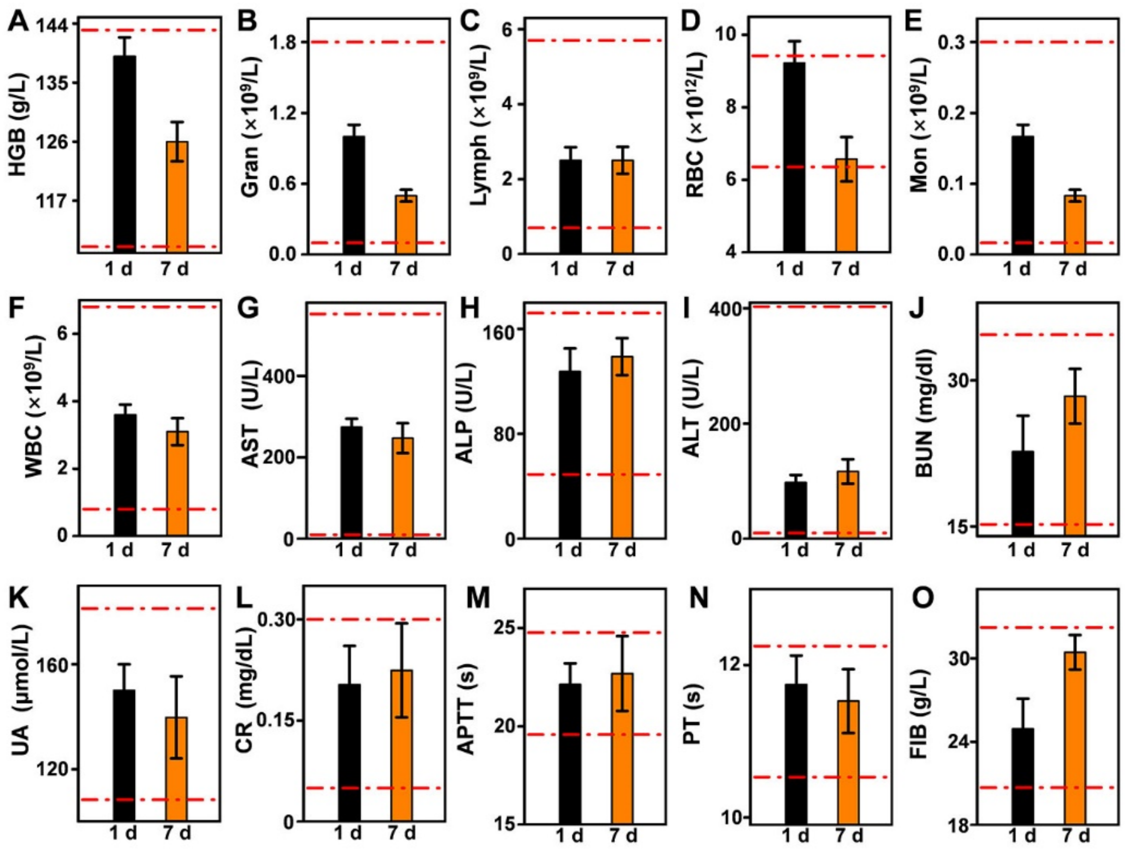

Figure S5. (A)–(O) Blood biochemistry parameters of BALB/c mice treated with Er-DCNPs. All of these parameters were within the normal range (red dotted line). The data were collected at different time point after intravenous injection: (A) Hemoglobin (HGB). (B) neutrophils (Gran). (C) lymphocyte (Lymph). (D) red blood cells (RBC). (E) monocytes (Mon). (F) white blood cells (WBC). (G) aspartate aminotransferase (AST). (H) alkaline phosphatase (ALP). (I) alanine aminotransferase (ALT). (J) blood urea nitrogen (BUN). (K) urea (UA). (L) creatinine (CR). (M) activated partial thromboplastin time (APTT). (N) prothrombin time (PT). (O) fibrinogen (FIB).

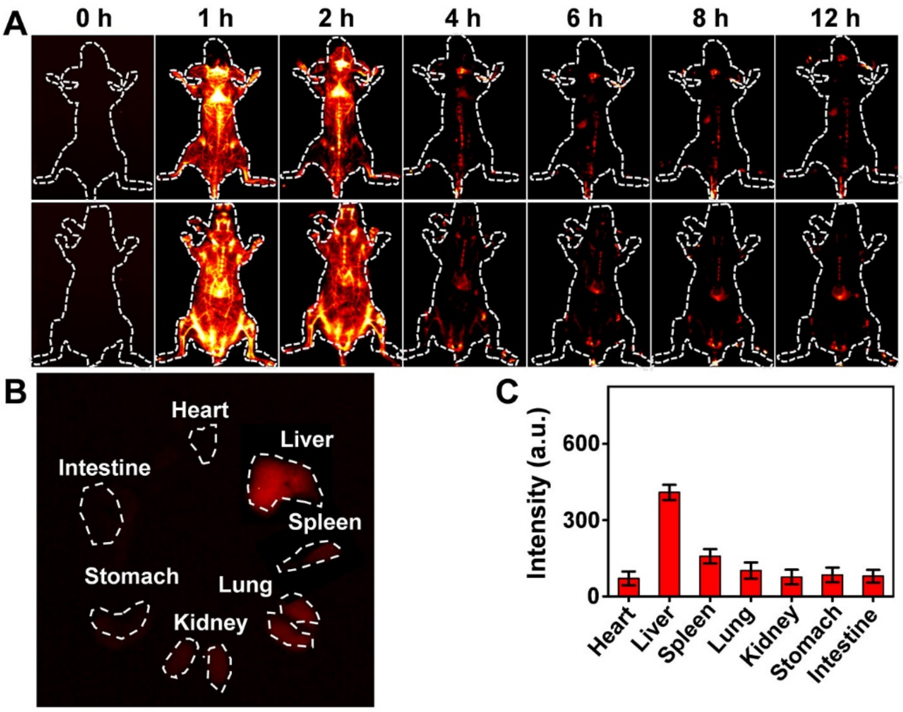

Figure S6. (A) The NIR-IIb bioimaging of whole-body in both prone and supine positions of the normal mouse at different times after intravenous injection of Er-DCNPs (n = 3). (B) *Ex vivo* fluorescence imaging of major organs at 12 h post-injection of Er-DCNPs. (C) Signal intensities of Er-DCNPs from different organs at 12-h post injection.

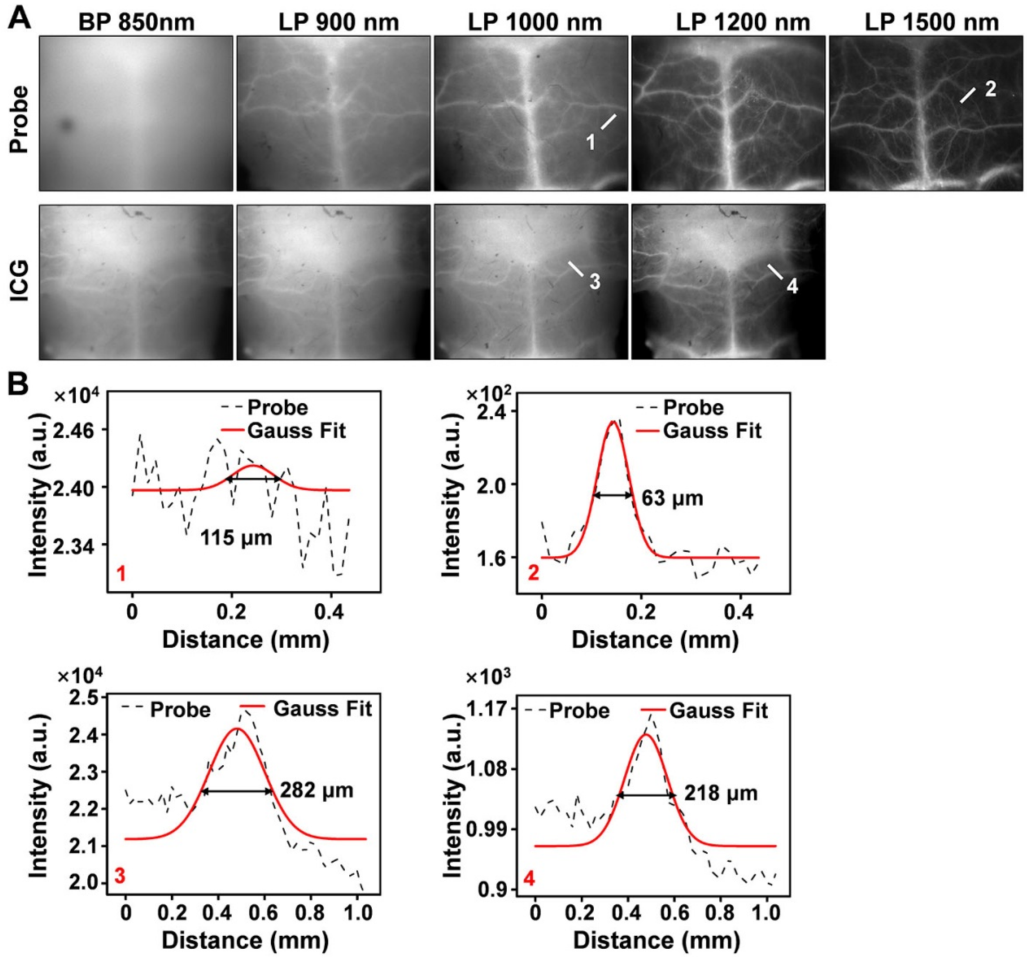

Figure S7. (A) Non-invasive NIR-IIb fluorescence images of cerebral vessels in C57BL/6 female mice under different filters after intravenous injection of Er-DCNPs and ICG (n = 3). (B) The fluorescence intensity profiles (dashed black line) and Gaussian fitting curve (solid red line) along the white lines 1-4 in the NIR-IIb fluorescence image shown in (A).

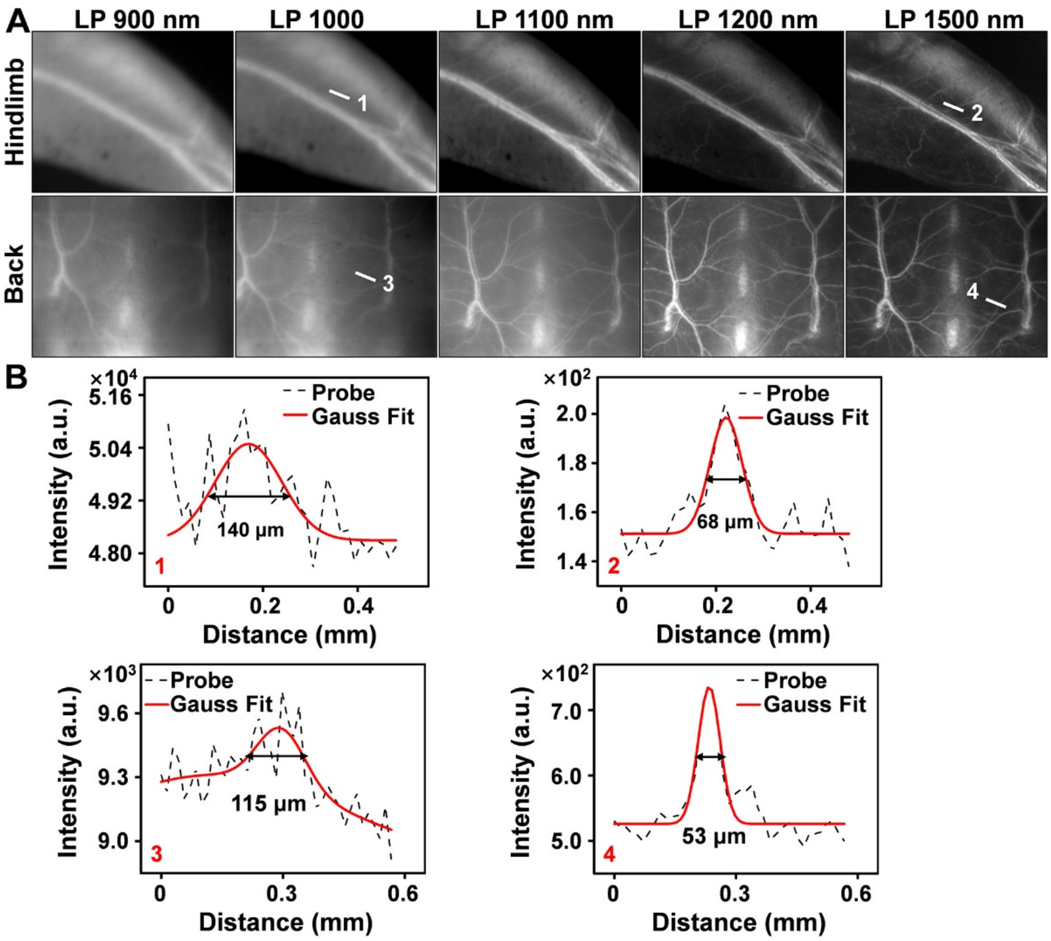

Figure S8. (A) Non-invasive NIR-IIb fluorescence images of hindlimb and back in C57BL/6 female mice under different filters after intravenous injection of Er-DCNPs (n = 3). (B) The fluorescence intensity profiles (dashed black line) and Gaussian fitting curve (solid red line) along the white lines 1-4 in the NIR-IIb fluorescence image shown in (A).

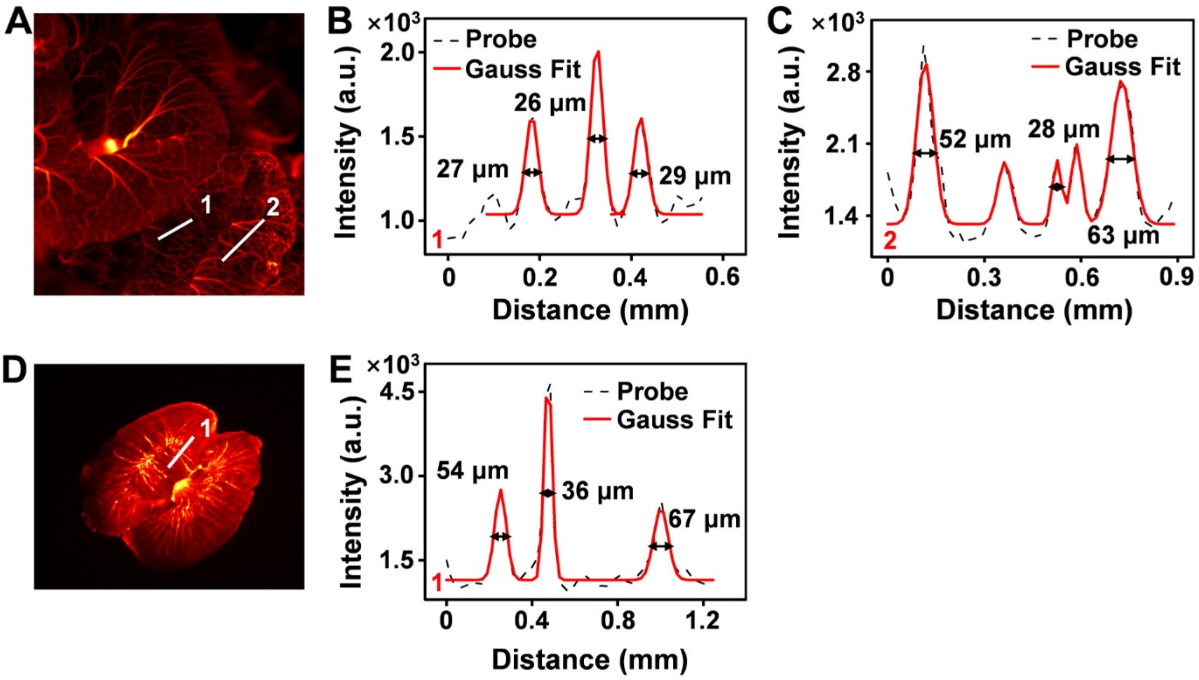

Figure S9. (A) NIR-IIb bioimaging of mice intestine after intravenous injection of Er-DCNPs. (B) The fluorescence intensity profiles (dashed black line) and Gaussian fitting curve (solid red line) along the white line 1 in the NIR-IIb fluorescence image shown in (A). (C) The fluorescence intensity profiles (dashed black line) and Gaussian fitting curve (solid red line) along the white line 2 in the NIR-IIb fluorescence image shown in (A). (D) NIR-IIb bioimaging of mice coronal section of left kidney after intravenous injection of Er-DCNPs. (E) The fluorescence intensity profiles (dashed black line) and Gaussian fitting curve (solid red line) along the white line 1 in the NIR-IIb fluorescence image shown in (D).

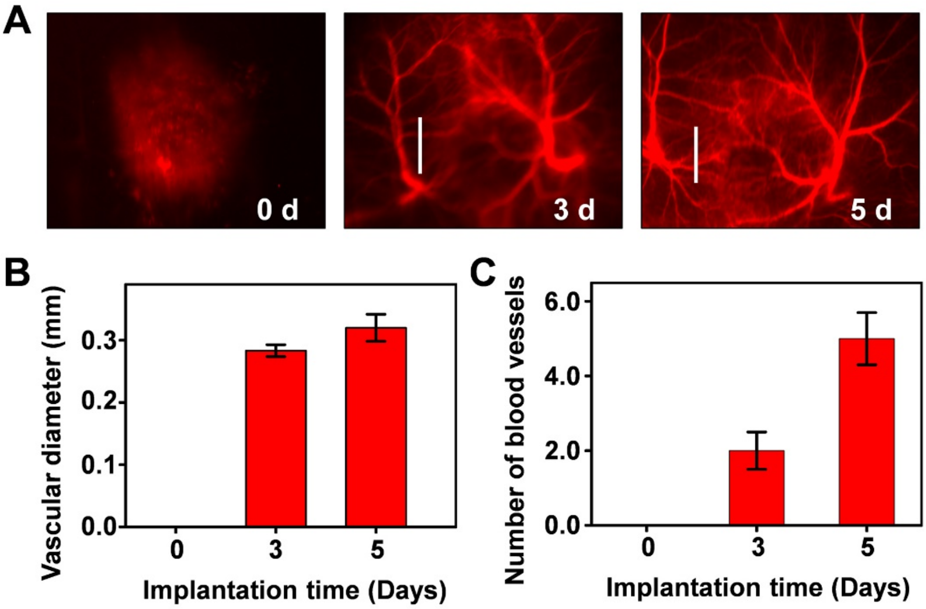

Figure S10. (A) The NIR-IIb imaging of the tumor at different days. (B) The vascular diameter of tumor vessels at different days along the white lines in the NIR-IIb fluorescence image shown in (A). (C) The number of tumor vessels at different days along the white lines in the NIR-IIb fluorescence image shown in (A).

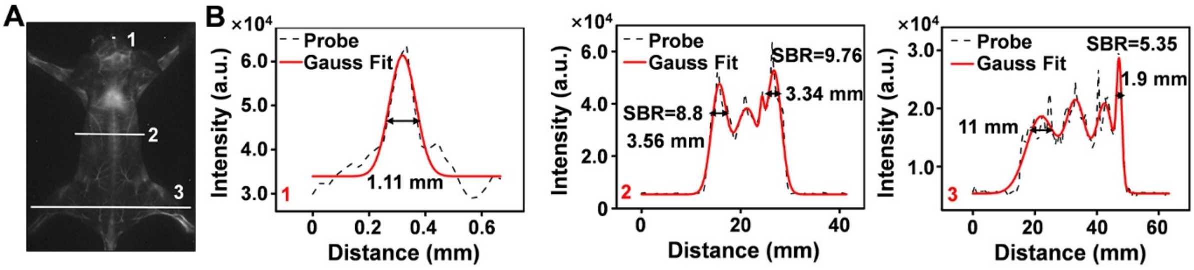

Figure S11. (A) The NIR-IIb fluorescence images of the mouse back vessels. (B) The fluorescence intensity profiles (dashed black line) and Gaussian fitting curve (solid red line) along the white lines 1-3 in the NIR-IIb fluorescence image shown in (A).

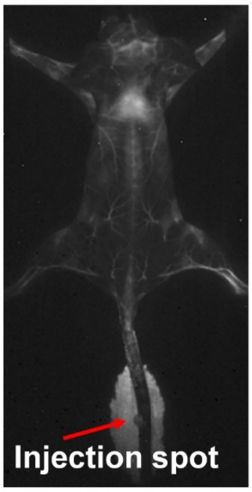

Figure S12. The NIR-IIb fluorescence images of leakage of tail-vein injected probe.

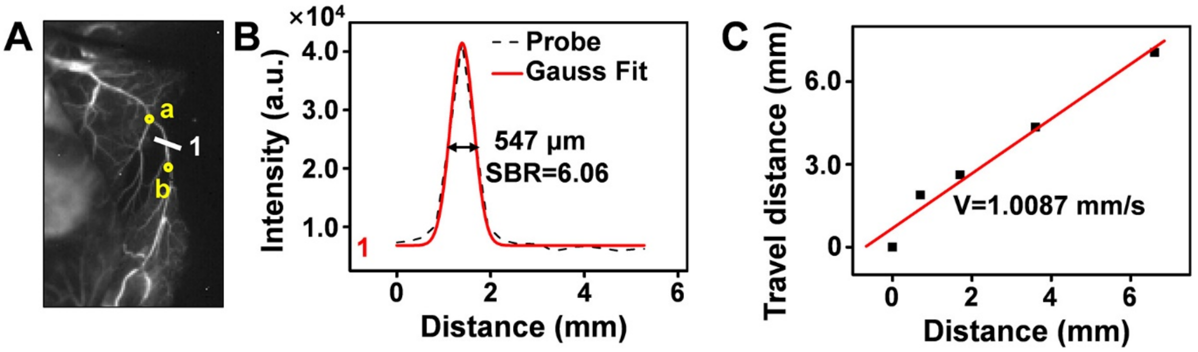

Figure S13. (A) High-magnification of vessel. (B) The fluorescence intensity profiles (dashed black line) and Gaussian fitting curve (solid red line) along the white line 1 in the NIR-IIb fluorescence image shown in (A). (C) Blood flow velocity (BFV) from a to b in (A). The slope of the function was calculated as BFV.

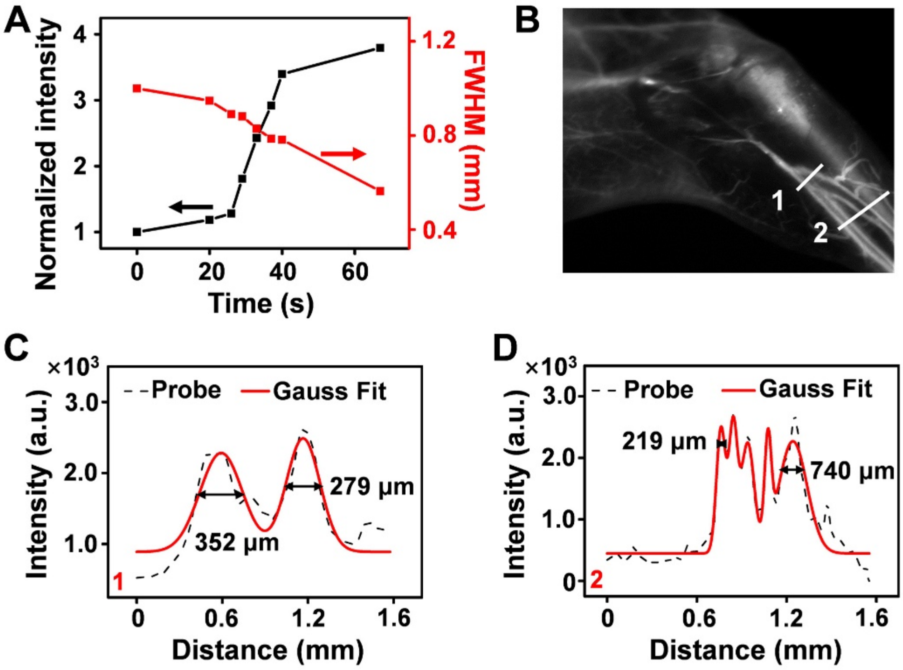

Figure S14. (A) The intensity (black line) and FWHM (red line) of thrombosis area. (B) The NIR-IIb fluorescence images of femoral artery after thrombolysis. (C)–(D) The fluorescence intensity profiles (dashed black line) and Gaussian fitting curve (solid red line) along the white lines 1 and 2 in the NIR-IIb fluorescence image shown in (B).

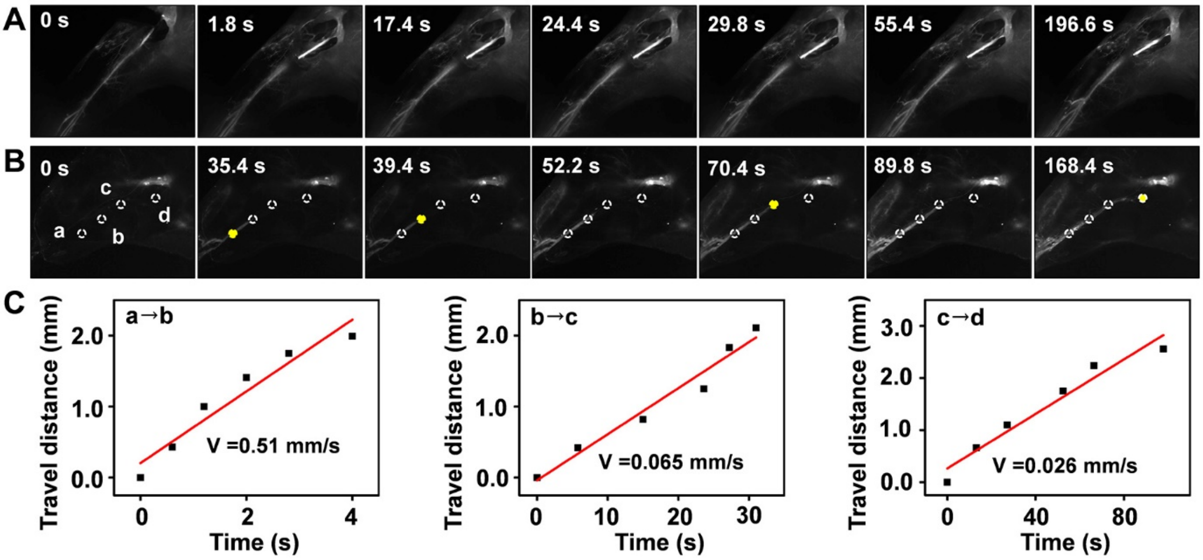

Figure S15. (A) Real-time NIR-IIb imaging of blood reperfusion process with Er-DCNPs after 40-min clipping. (B) Real-time NIR-IIb imaging of blood reperfusion process with Er-DCNPs after 1.5 h clipping. Four regions of interest (ROI) (white circles) selected from the femoral artery (named as a, b, c and d) to evaluate the ischemic reperfusion process. Yellow fill indicates the flow front. (C) The ischemic perfusion rate of the femoral artery from a to b, b to c and c to d in (B), respectively. The slope of the function was calculated as BFV.

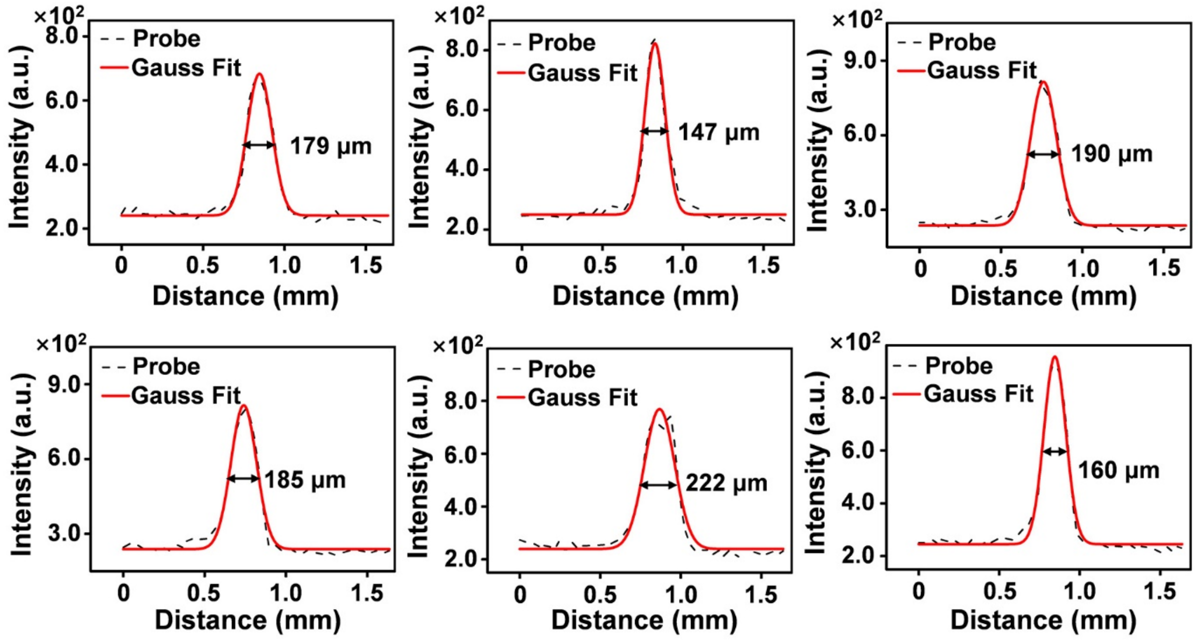

Figure S16. The fluorescence intensity profiles (dashed black line) and Gaussian fitting curve (solid red line) in the NIR-IIb fluorescence image shown in Figure S15B, from a to b.

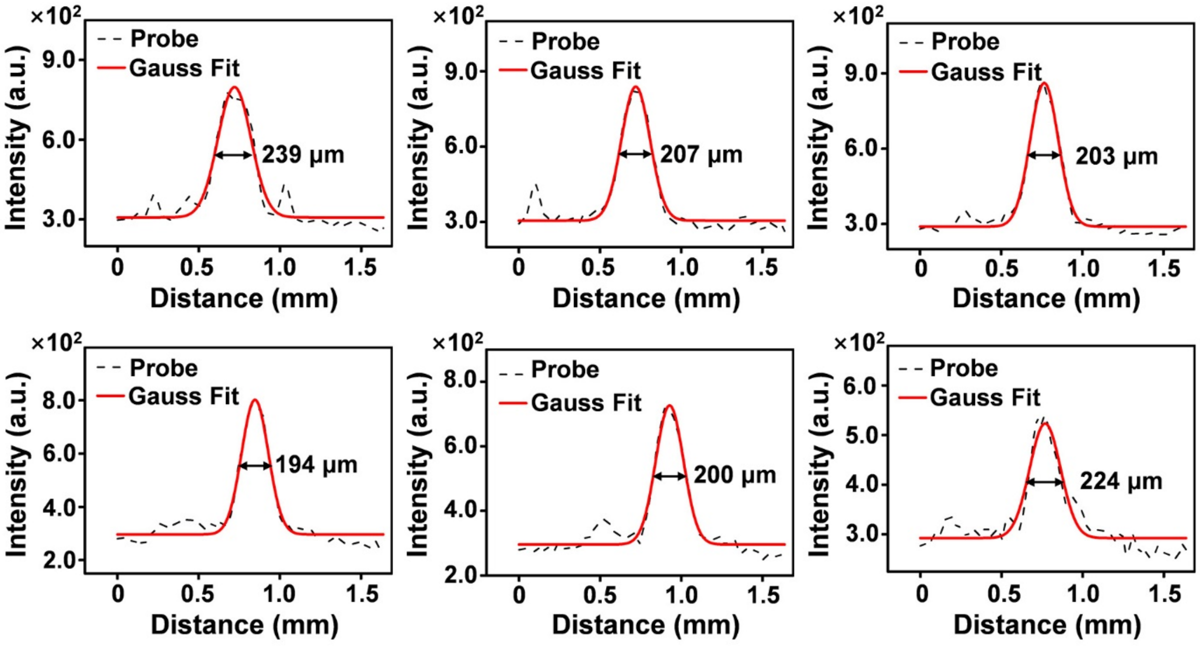

Figure S17. The fluorescence intensity profiles (dashed black line) and Gaussian fitting curve (solid red line) in the NIR-IIb fluorescence image shown in Figure S15B, from b to c.

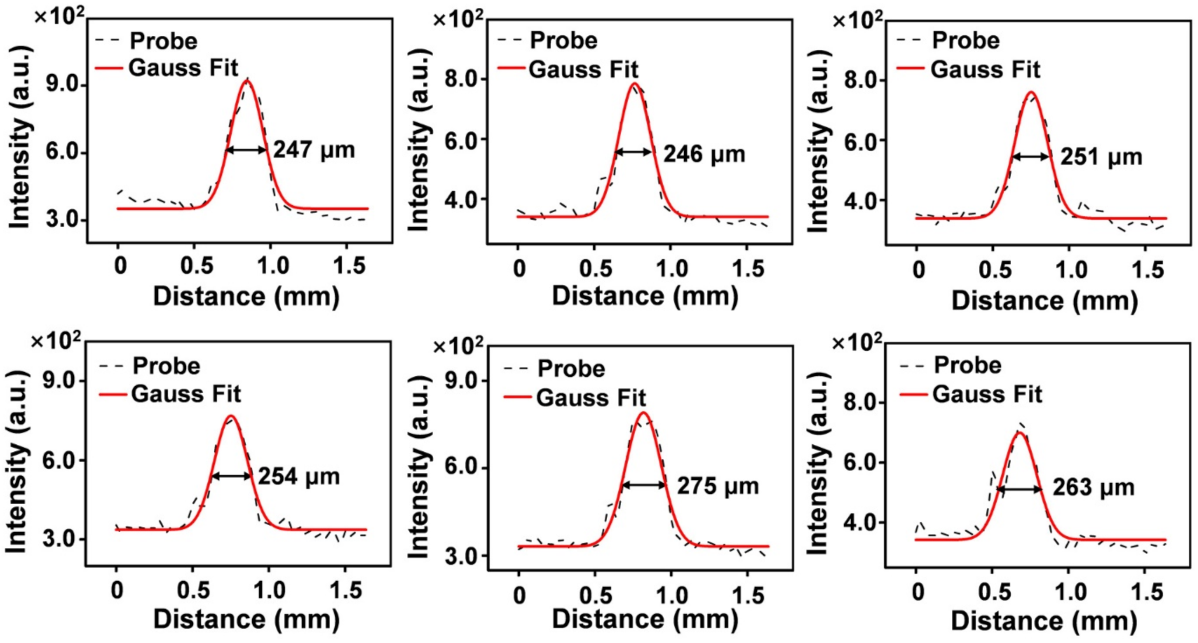

Figure S18. The fluorescence intensity profiles (dashed black line) and Gaussian fitting curve (solid red line) in the NIR-IIb fluorescence image shown in Figure S15B, from c to d.

Table S1. The average diameters of blood vessel in Figure S15B.

Interval			FWHM				Average
a→b	160 μm	222 μm	185 μm	190 μm	147 μm	179 μm	180.5 μm
b→c	224 μm	200 μm	194 μm	203 μm	207 μm	239 μm	211.2 μm
c→d	263 μm	275 μm	254 μm	251 μm	246 μm	247 μm	256 μm
